# E–H Bond Activation of Ammonia and Water by a Geometrically Constrained Phosphorus(III) Compound

**DOI:** 10.1002/anie.201506998

**Published:** 2015-09-22

**Authors:** Thomas P Robinson, Daniel M De Rosa, Simon Aldridge, Jose M Goicoechea

**Affiliations:** Department of Chemistry, University of Oxford, Chemistry Research Laboratory 12 Mansfield Road, Oxford, OX1 3TA (UK) E-mail: simon.aldridge@chem.ox.ac.uk jose.goicoechea@chem.ox.ac.uk

**Keywords:** ammonia activation, main group elements, phosphorus, tridentate ligands, water activation

## Abstract

The synthesis of a phosphorus(III) compound bearing a *N*,*N*-bis(3,5-di-*tert*-butyl-2-phenoxy)amide ligand is reported. This species has been found to react with ammonia and water, activating the E–H bonds in both substrates by formal oxidative addition to afford the corresponding phosphorus(V) compounds. In the case of water, both O–H bonds can be activated, splitting the molecule into its constituent elements. To our knowledge, this is the first example of a compound based on main group elements that sequentially activates water in this manner.

The controlled activation of polar small molecules, such as ammonia and water, is a challenging and highly desirable target, as these substrates are abundant and inexpensive feedstocks for the synthesis of value-added chemicals and the generation of renewable energy.[1, 2] The processes required to carry out such transformations (for example, oxidative addition and reductive elimination) are most commonly associated with precious-metal catalysts, for which crustal abundance, expense, and toxicity are significant issues. Consequently, the last decade has seen significant advances in the development of main group species that are capable of activating a number of challenging small-molecule substrates.[3] Such systems include alkyl(amino) carbenes (AACs),[4] low-oxidation-state compounds of the heavier Group 13 and 14 elements,[5, 6] and frustrated Lewis pairs (FLPs).[7] These species have been shown to activate substrates including ammonia and/or dihydrogen; several such systems have even demonstrated the ability to carry out these processes catalytically.[8] New routes resulting in the activation of N–H bonds in ammonia are particularly appealing because of the dearth of transition-metal systems capable of effecting such a transformation[9–21] and the relevance of N–H activation to a number of potentially important industrial processes.[22]

In addition to these high profile examples, recent studies have shown that complexes of phosphorus(III) with distorted geometry can cleave a range of polarized E–H bonds (E=O or N). Studies by Arduengo et al. and Radosevich and co-workers have shown that the planar T-shaped phosphorus system, **A**, facilitates the oxidative addition of ammonia, primary amines, and alcohols (Figure 1).[23, 24] Furthermore, **A** can be used in conjunction with ammonia–borane to facilitate the catalytic reduction of azobenzene.[25] Related studies have since extended this approach to nonplanar phosphorus triamide (**B**) and diazadiphosphapentalene (**C**),[26, 27] both of which activate E–H bonds through a ligand-assisted mechanism.

**Figure 1:**
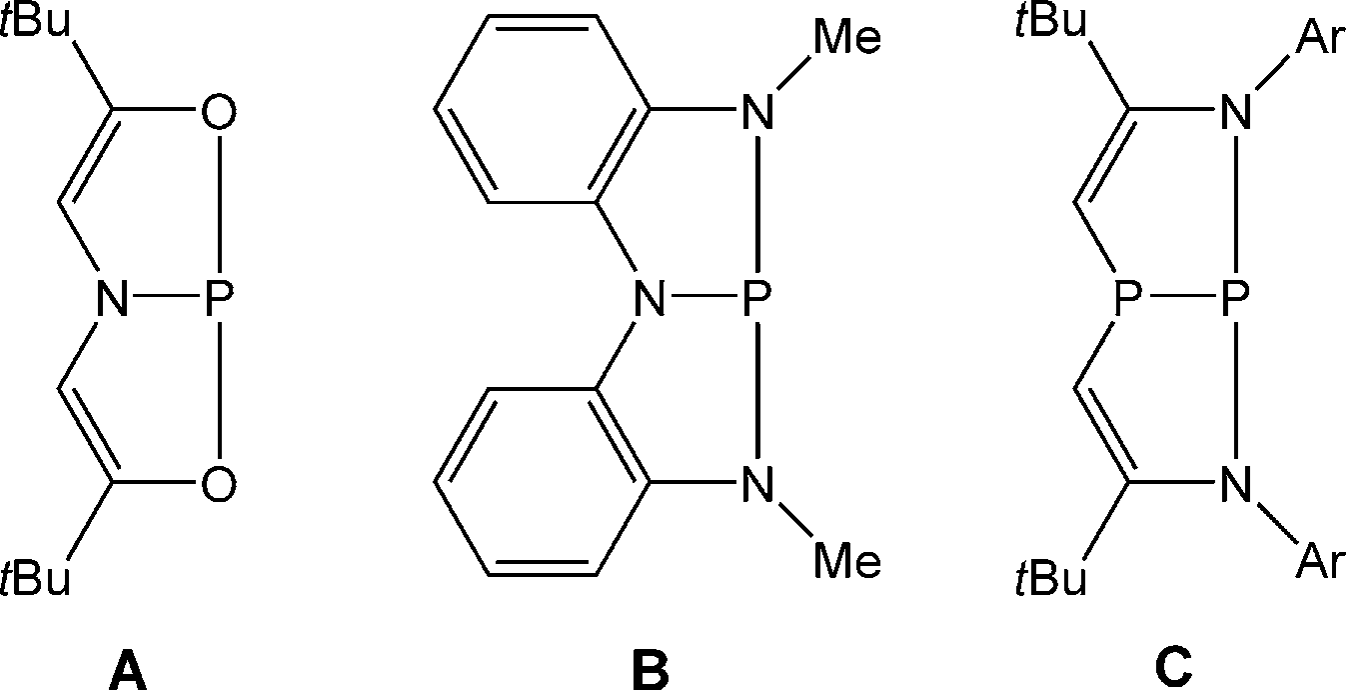
Structures of three geometrically constrained phosphorus(III) species.

The mechanisms by which **A**–**C** activate small molecules are strongly dependent on the sterics/electronics of the ligand backbone. For example, it has been shown that, following the activation of alcohols by **A**, proton migration to the ligand backbone occurs, regenerating a phosphorus(III) compound.[23] This inspired us to investigate the utility of the tridentate *N*,*N*-bis(3,5-di-*tert*-butyl-2-phenoxy)amide ligand (denoted [ONO]^3−^), previously studied with transition-metal centers,[28] as a support for a novel geometrically constrained phosphorus system. It was hypothesized that the aromatic backbone of ONO^3−^ should preclude proton migration to the ligand following E–H bond activation.

The reaction between H_3_[ONO], PCl_3_, and triethylamine (Scheme 1) leads to the quantitative formation of a single product (**1**). A singlet for the product was detected in the ^31^P NMR spectrum at *δ*=168.6 ppm.[29] This chemical shift falls within the characteristic range exhibited for compounds **A**, **B**, and **C** (*δ*=187.0, 159.8 and 173.2 ppm, respectively). Additionally, ^1^H NMR analysis confirmed that the molecule had a symmetric ligand environment (see the Supporting Information for full experimental details).

**Scheme 1:**
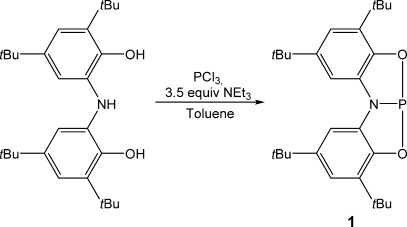
Synthesis of 1.

**1** was isolated as a compositionally pure colorless solid, solutions of which turn violet upon exposure to oxygen, presumably because of the redox-active nature of the ONO^3−^ ligand. Crystals of **1** suitable for single-crystal X-ray diffraction studies were grown from cold pentane and the molecular structure confirmed the formation of the desired phosphorus(III) species (Figure 2).

**Figure 2:**
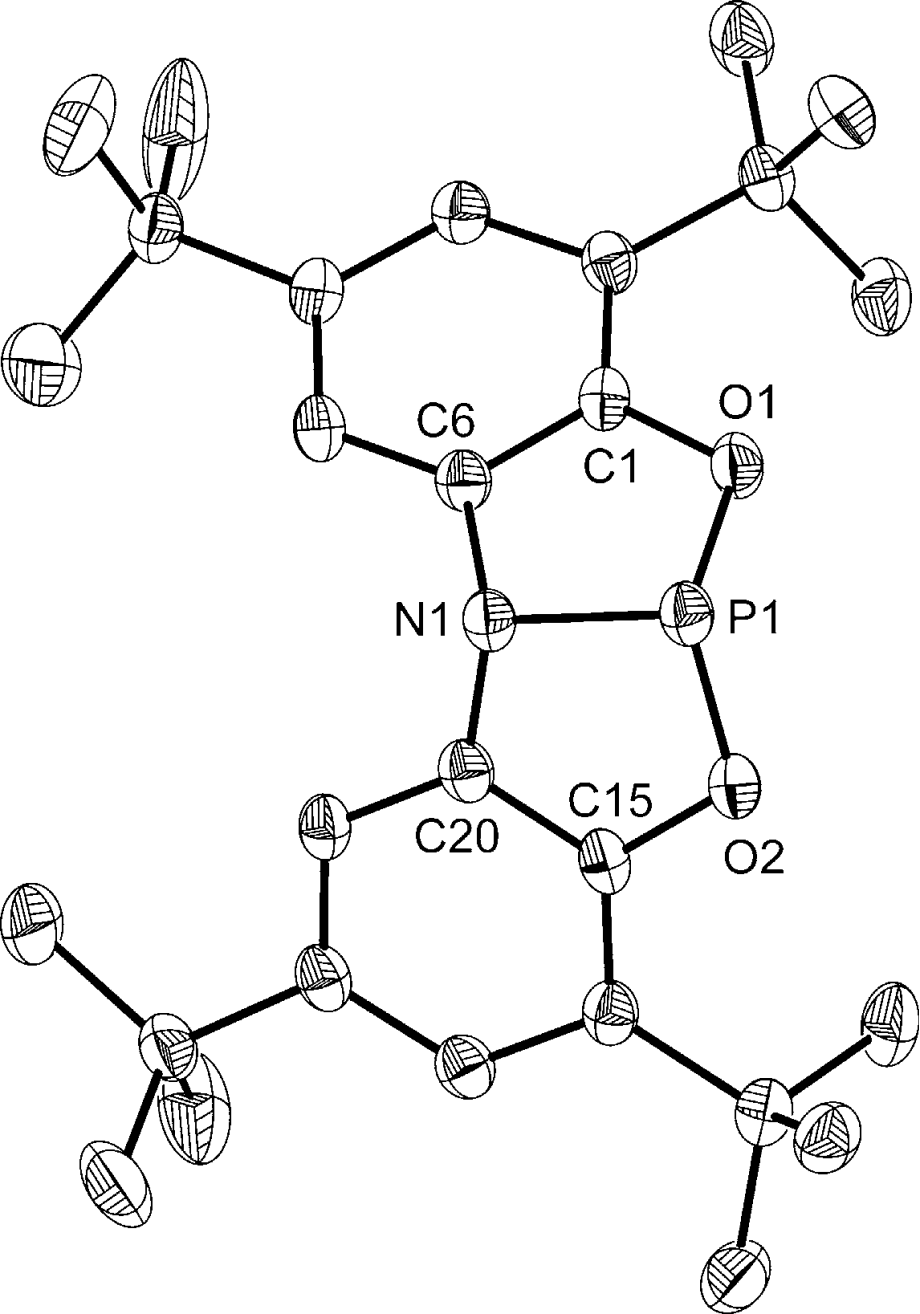
Molecular structure of 1 (thermal ellipsoids set at 50 % probability; hydrogen atoms are omitted for clarity).[29] Selected bond lengths [Å] and angles [°]: P1–N1 1.757(1), P1–O1 1.659(1), P1–O2 1.652(1); O1-P1-O2 109.55(5), N1-P1-O1 93.21(5), N1-P1-O2 93.36(5), C6-N1-C20 115.67(10), C6-N1-P1 107.80(8), C20-N1-P1 108.09(8).

The molecular structure of **1** differs from the planar geometry exhibited by the analogous 10-P-3 species **A** (where 10-P-3 denotes a 3-coordinate P center with 10 valence electrons) with pyramidalization at both nitrogen and phosphorus centers providing pseudo *C_s_* symmetry, similar to **B** and **C**. Whilst the O-P-N bond angles (93.21(5)° and 93.36(5)°) are statistically identical, the O-P-O angle is significantly larger because of the constraints of the ligand backbone (109.55(5)°). The sum of the bond angles around the phosphorus center (296.12°) thus confirms the nonplanar geometry. The P–N bond length of **1** (1.757(1) Å) is the same (within error) as that of **B** (1.761(1) Å) but is significantly longer than that of **A** (1.703(2) Å), suggesting negligible donation of the nitrogen lone pair to the orbitals on phosphorus. The deviation from planarity also results in a shortening of the P–O bonds of **1** (1.659(1) Å and 1.652(1) Å) relative to **A** (1.835(2) Å and 1.792(2) Å), which now fall within the expected range for P^III^–O bonds. Calculations using density functional theory (DFT) reveal that pyramidal (*C_s_*) and planar (*C*_2*v*_) isomers of **1** are within 4 kJ mol^−1^ of one another, well within the error of the calculations. This suggests that fluxionality between isomers is likely to be present in solution. The calculations also reveal an energetically accessible LUMO for the optimized *C*_2*v*_ geometry which is predominantly composed of phosphorus atomic orbital character (54.7 %). This implies that that subsequent reactions involving the activation of small molecules might proceed via an initial nucleophilic pathway, corroborating experimental findings that **1** does not react with non-nucleophilic substrates such as phenylacetylene and phenylsilane (see below).

The activation of the N–H bonds of ammonia has long posed a significant challenge for transition-metal complexes, on account of the unfavorable coordination/activation equilibrium for this substrate. For this reason examples are scarce, and even fewer constitute classical oxidative additions.[9–21] Therefore, advances in main group mediated activation of ammonia are of great interest.[4, 5, 6c–d, 24, 26, 27, 30–32] The reaction of **1** with ammonia gas (1 atm) results in the quantitative conversion of **1** to a new product, **2** (Scheme 2). The ^31^P NMR spectrum of **2** exhibits a resonance at *δ*=−46.1 ppm as a doublet of triplets (^1^*J*_P–H_=819 Hz, ^2^*J*_P–H_=11 Hz), consistent with the oxidative addition of a single N–H bond at the phosphorus center. Furthermore, complementary doublets are detected in the ^1^H NMR spectrum at *δ*=8.46 ppm and *δ*=2.16 ppm integrating to one (P*H*) and two protons (N*H*_2_), respectively. The proposed structure of **2** was confirmed through single-crystal X-ray diffraction studies (Figure 3). Although ammonia has been shown to react with both **A** and **B**[24, 26] in a process that constitutes a formal oxidative addition, **2** is the first structurally authenticated example of such a compound.

**Figure 3:**
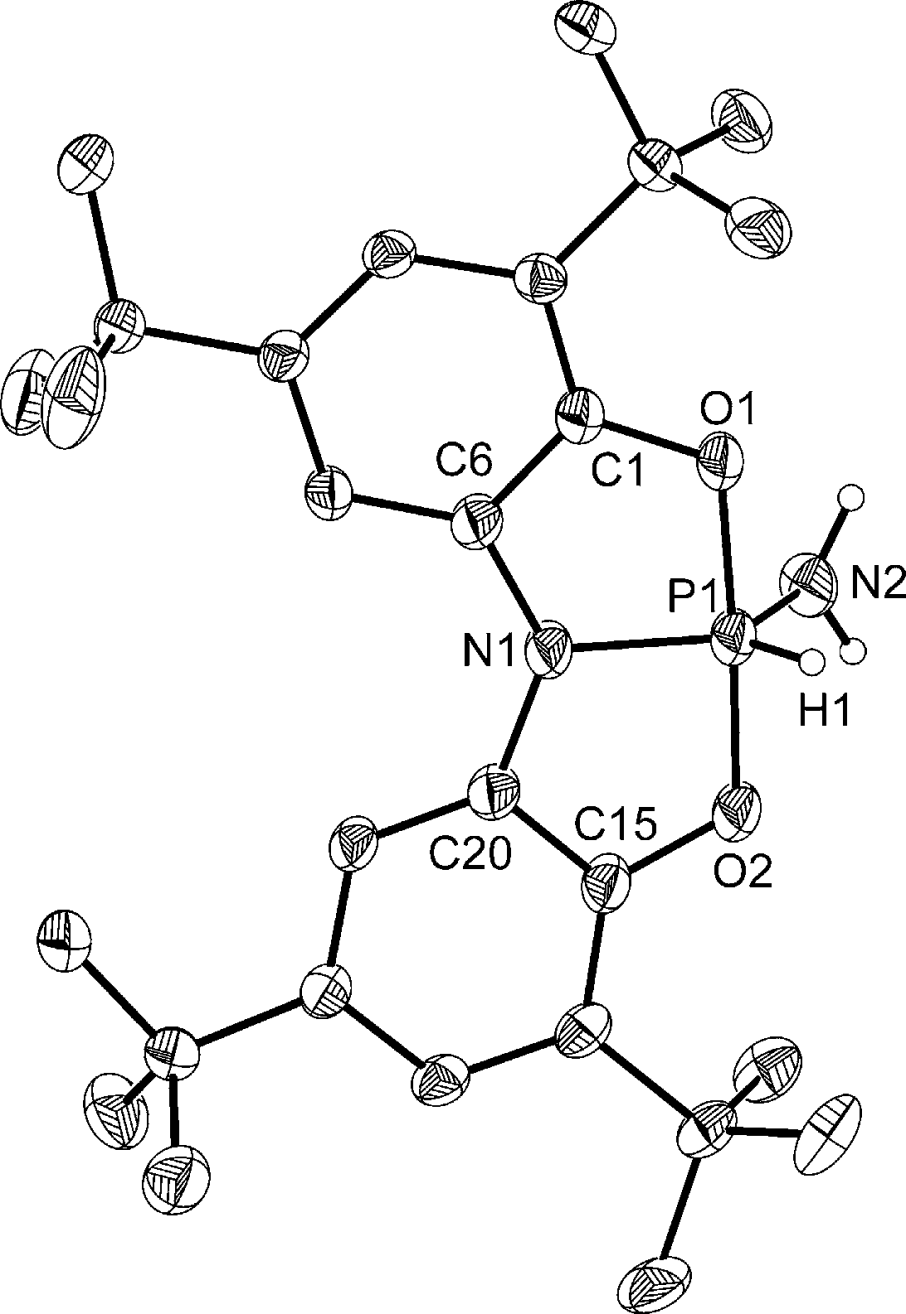
Molecular structure of one the two independent molecules of 2 in the asymmetric unit (thermal ellipsoids set at 50 % probability; all hydrogen atoms, with the exception of those bonded to phosphorus and nitrogen, are omitted for clarity).[29] Despite the substitutional disorder between the hydride (H1) and amide (N2H_2_) moieties we were able to locate the proton positions in the difference Fourier map, however the bond lengths needed to be restrained. Selected bond lengths [Å] and angles [°] of one of the independent molecules within the crystal lattice: P1–N1 1.700(2), P1–O1 1.718(2), P1–O2 1.710(2); O1-P1-O2 176.67(11), N1-P1-O1 88.64(11), N1-P1-O2 88.36(11).

**Scheme 2:**
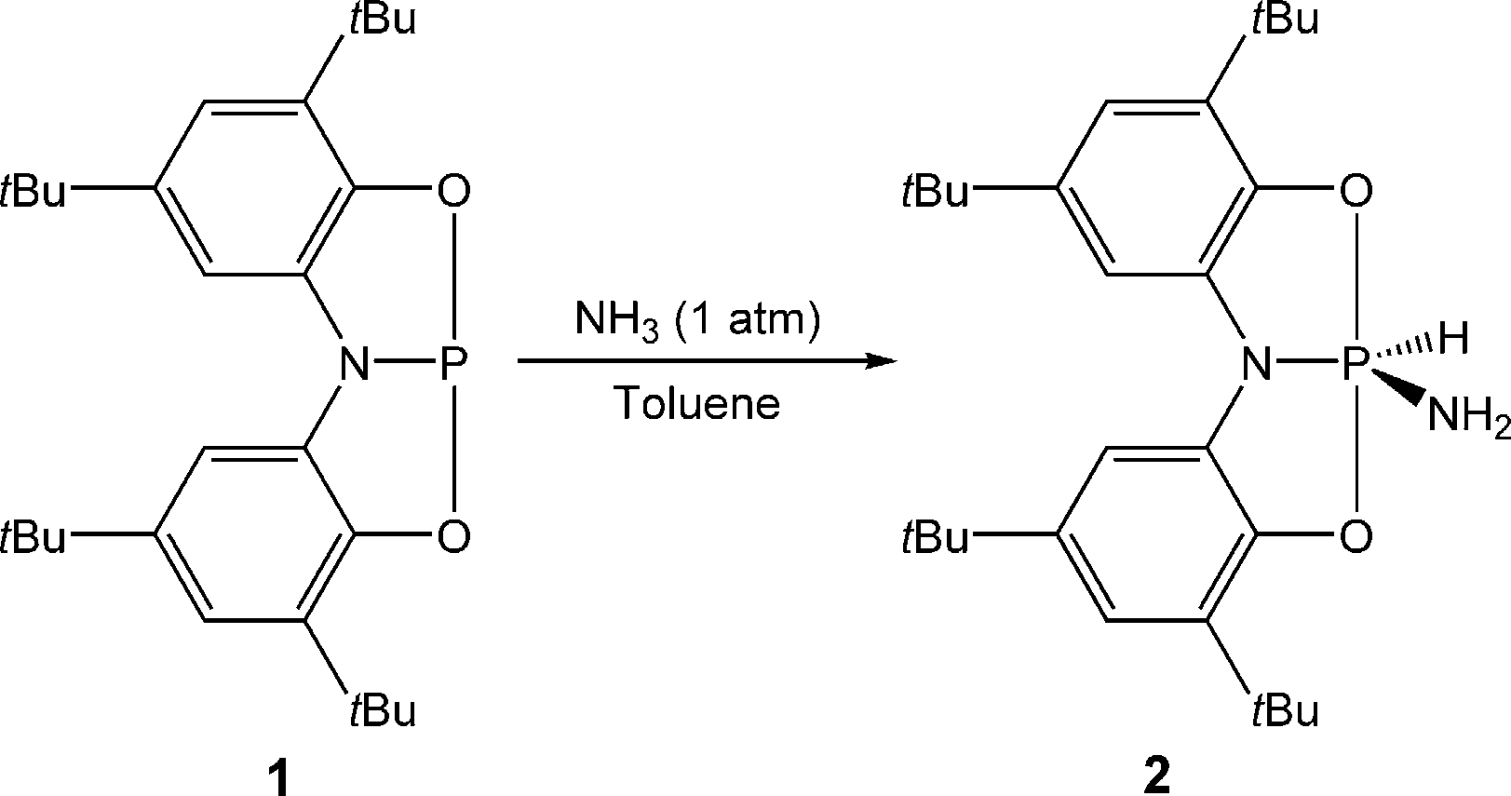
The reaction of 1 with NH_3_ to form 2.

The molecular structure of **2** exhibits a trigonal-bipyramidal structure, in which the hydride and two amide moieties adopt equatorial positions. The ONO^3−^ ligand adopts a planar conformation, analogous to the mesitylamine activation product of **A**, [**A**(H)(NHMes)].[24] By contrast, the reaction products of **B** have been shown to retain a folded ligand geometry.[26] The axial disposition of the P–O bonds of **2** results in their lengthening relative to the analogous bonds in **1**, whilst the increased valency effects a contraction of the P1–N1 bond length (average value of 1.701 Å). Substitutional disorder of the amide and hydride substituents over the two equatorial sites precludes the analysis of the relevant bond metrics.

The facile activation of ammonia by **1** encouraged us to explore its reactivity towards other more challenging substrates. Remarkably, it was discovered that **1** undergoes rapid addition of water at the phosphorus center to form **3**, with the reaction proceeding quantitatively in the presence of a single equivalent of substrate (Scheme 3). The ^31^P NMR spectrum of the reaction mixture shows a broad doublet resonance at *δ*=−36.9 ppm (^1^*J*_P–H_=881 Hz), which collapses to a singlet upon proton decoupling. The signal for the corresponding hydride is detected as a doublet in the ^1^H NMR spectrum at *δ*=8.22 ppm whilst a broad resonance at *δ*=3.87 ppm is evidence of the hydroxyl proton. Furthermore, FTIR spectral analysis of the product reveals a broad band at 3425 cm^−1^, consistent with an O–H bond stretch. Crystals of **3** were grown from a concentrated toluene solution and single-crystal X-ray diffraction analysis confirmed the oxidative addition of water over the phosphorus center (Figure 4). This observation comes in stark contrast to the reactivity of **A**, which is reported to hydrolyze to the corresponding free ligand and phosphoric acid.[23]

**Figure 4:**
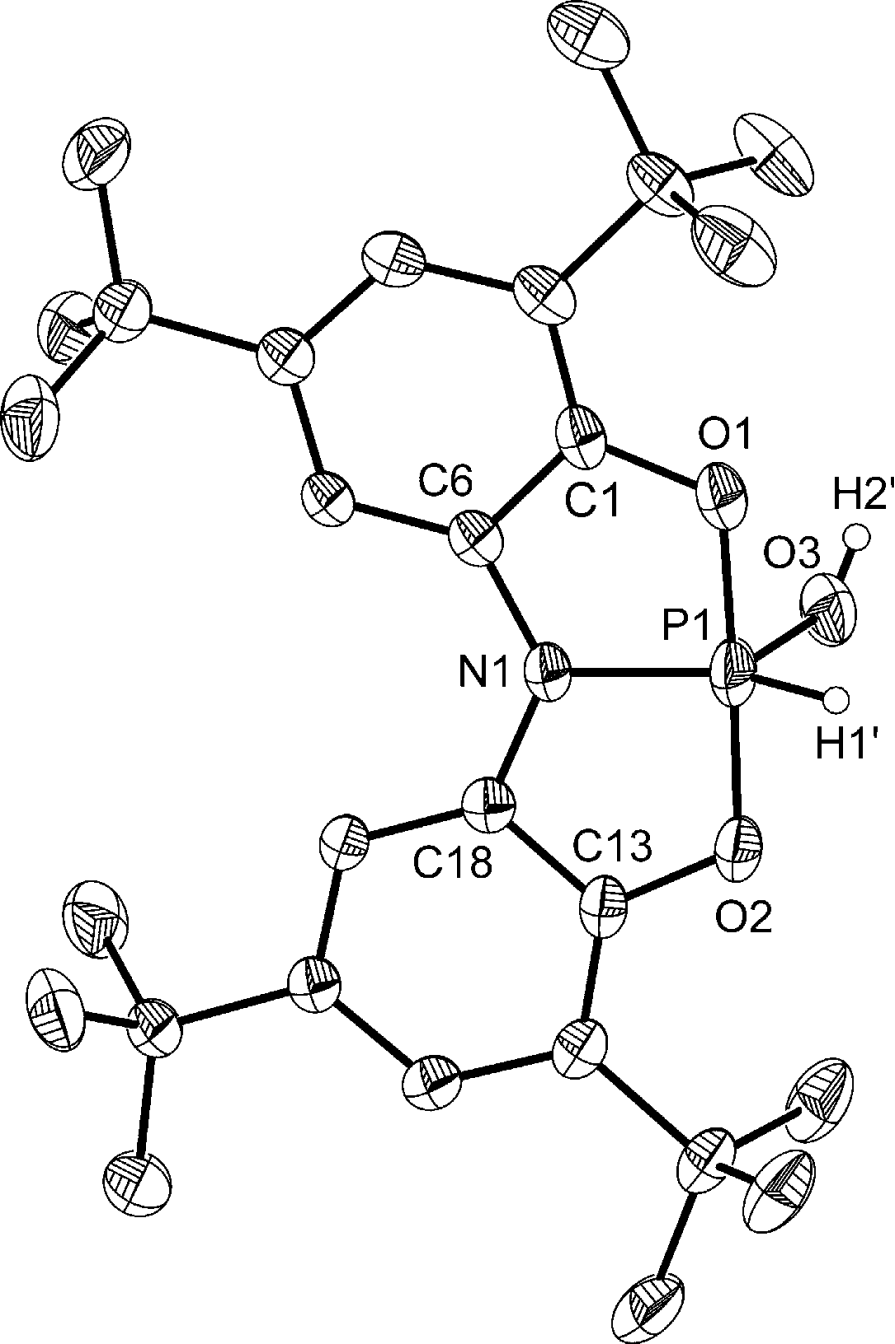
Molecular structure of 3 (thermal ellipsoids set at 50 % probability; all hydrogen atoms, with the exception of those bonded to phosphorus and oxygen, are omitted for clarity).[29] Selected bond lengths [Å] and angles [°]: P1–N1 1.690(2), P1–O1 1.681(2), P1–O2 1.689(2), P1–O3 1.774(3), P1–H1 1.23(3); O1-P1-O2 177.90(8), O1-P1-O3 89.58(11), O2-P1-O3 91.15(11), N1-P1-O1 89.13(8), N1-P1-O2 88.77(8), N1-P1-O3 110.58(8), O1-P1-H1 92.2(15), O2-P1-H1 89.6(15), O3-P1-H1 103(2), N1-P1-H1 146.8(16).

**Scheme 3:**
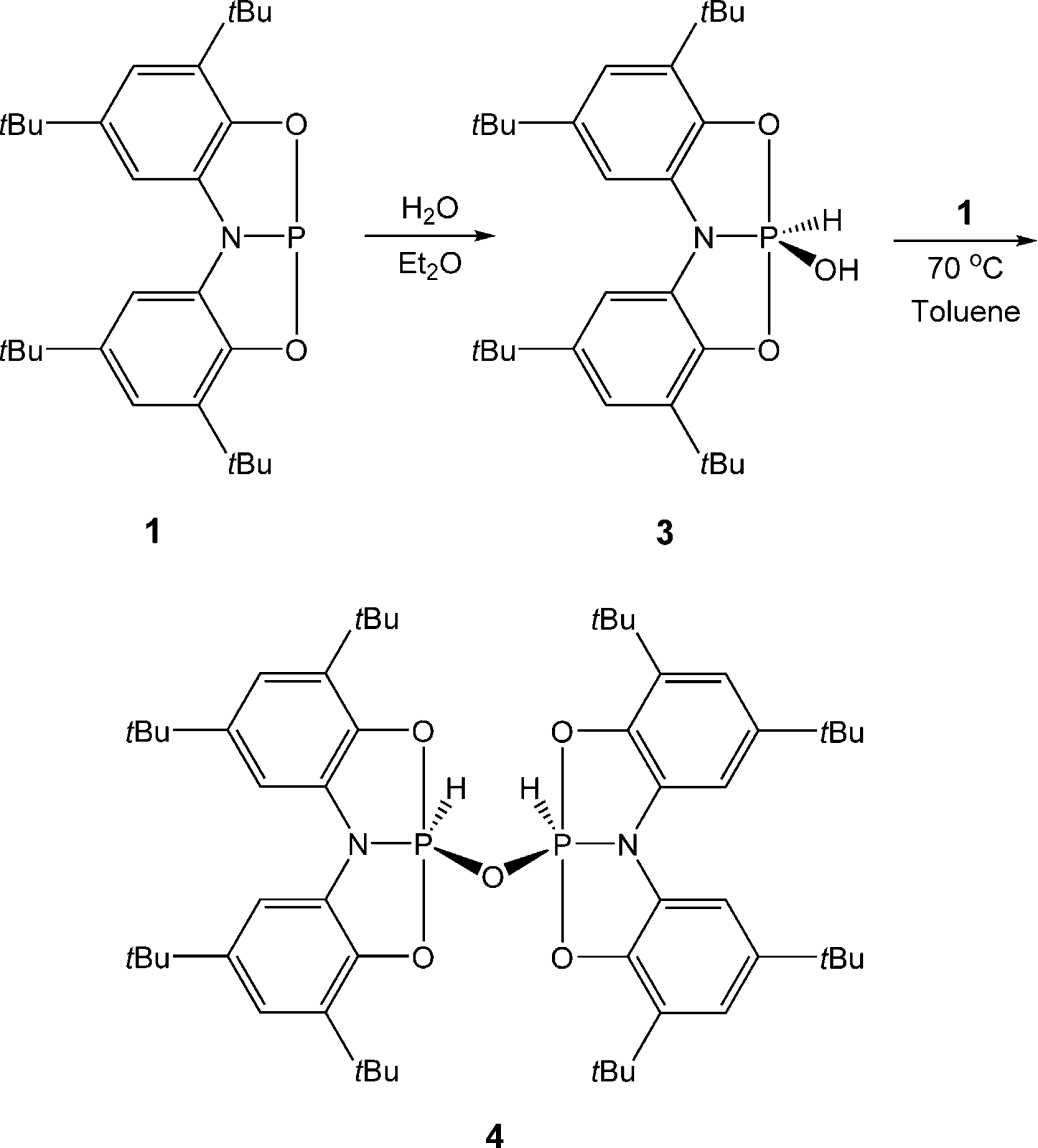
Stepwise oxidative addition of water by 1 to form 3 and 4.

The oxidative activation of water is of immense current interest, representing a crucial step in metal-catalyzed water splitting. Despite this fact, there are few transition-metal complexes that are able to effect this transformation.[2] Although there are several reports outlining similar reactivity at main group centers,[33–41] there are currently only two examples that are able to do so without the use of forcing conditions, non-equimolar loadings of water, or secondary activating substrates.[37, 41]

The room-temperature reaction of **3** with one equivalent of deuterium oxide results in exchange of the hydroxyl proton. Interestingly, when the reaction is carried out at 70 °C, exchange of the phosphorus-bound hydride is also observed, providing a 1:1 isotopic distribution. The relative ease of H/D exchange is in agreement with a previous report by Pörschke et al.[35] In addition to the previously observed doublet, attributable to the phosphorus center of **3**, the ^31^P NMR spectrum of the reaction mixture also shows a triplet at *δ*=−37.7 ppm (^1^*J*_P–D_=133 Hz). A complementary doublet is also observed in the ^2^D NMR spectrum at *δ*=8.75 ppm). The magnitude of the coupling constant is consistent with the difference in gyromagnetic ratio of the hydrogen and deuterium nuclei (γ_H_/γ_D_=6.5).

The lability of the hydroxyl proton of **3** encouraged us to investigate the equimolar reaction of this product with a second equivalent of **1** (Scheme 3). At room temperature, the reaction proceeds slowly, however, after 12 h at 70 °C, the ^31^P NMR spectrum of the reaction mixture indicates that **3** has been fully converted to a new product, **4**, which exhibits a second-order multiplet resonance at *δ*=−44.0 ppm (m, ^1^*J*_P–H_=913 Hz, ^2^*J*_P–P_=−30 Hz, ^3^*J*_P–H_=1 Hz), which collapses to a singlet on proton decoupling. A similar multiplet is also observed in the corresponding ^1^H NMR spectrum at *δ*=8.43 ppm. Single-crystal X-ray diffraction studies revealed that the product **4** featured an oxygen-bridged dimeric structure (Figure 5), arising from oxidative addition of the remaining O–H bond over the phosphorus(III) center of **1**, thereby completely splitting water into its constituent elements. Similarly, reaction of **1** with substoichiometric amounts of H_2_O also leads to the formation of **4**. Subsequent reaction of **4** with water regenerates the monomeric precursor **3**. To our knowledge, stepwise oxidative addition of water by a main group system has not previously been reported. However, similar reactivity has been postulated by Driess and co-workers who isolated a mixed-valence disiloxane from the reaction between water and a silylene, the synthesis of which was proposed to proceed via an unisolable [LSi(OH)(H)] intermediate.[36] Transition-metal complexes of silyenes are also know to react in such a manner.[42] Additionally, Bercaw and Hillhouse have reported the related Group 4 complexes [{Cp*_2_M(H)}_2_μ-O] (M=Zr and Hf; Cp*=pentamethylcyclopentadienyl), formed via σ-bond metathesis pathways.[10] It is worth noting at this stage that **2** does not react further with **1** to afford the related imide-bridged species.

**Figure 5:**
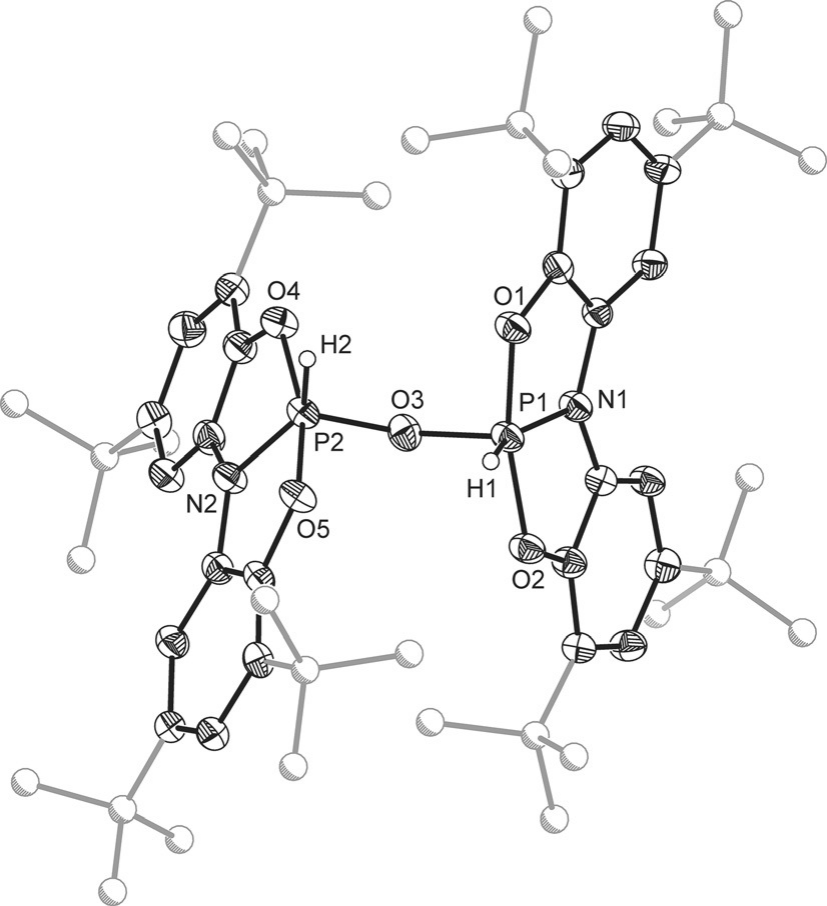
Molecular structure of one the two independent molecules of 4 in the asymmetric unit (thermal ellipsoids set at 50 % probability; all hydrogen atoms, with the exception of those bonded to phosphorus, are omitted for clarity).[29] Selected bond lengths for one of the two independent molecules in the asymmetric unit [Å] and angles [^o^]: P1–O1 1.693(2), P1–O2 1.680(2), P1–O3 1.628(2), P1–N1 1.710(2), P1–H1 1.30(3), P2–O3 1.601(2), P2–O4 1.680(2), P2–O5 1.692(2), P2–N2 1.707(2), P2–H2 1.24(3); P1-O3-P2 134.8(1), O1-P1-O2 166.0(1), O1-P1-O3 97.9(1), O1-P1-H1 87.4(12), O2-P1-O3 96.1(1), O2-P1-H1 88.7(12), O3-P1-H1 102.5(14), N1-P1-O1 89.0(1), N1-P1-O2 89.0(1), N1-P1-O3 102.2(1), N1-P1-H1 155.3(14); O4-P2-O5 163.7(1), O4-P2-O3 96.8(1), O4-P2-H2 88.6(16), O5-P2-O3 99.4(1), O5-P2-H2 84.7(16), O3-P2-H2 110.2(17), N2-P2-O3 106.0(1), N2-P2-O4 88.3(1), N2-P2-O5 88.4(1), N2-P2-H2 143.7(17).

The molecular structures of **3** and **4** are both significantly distorted from a trigonal-bipyramidal geometry about the phosphorus center with the geometry of the latter much closer to square-based pyramidal (*τ*=0.52 and 0.17–0.33, respectively). In both cases, the N-P-H bond angles are relatively large and the N-P-O_(OH/μ-O)_ angles small, due to a greater degree of negative hyperconjugation into the P–O_(OH/μ-O)_ σ* orbital than into the respective P–N σ* orbital of **2**. The chelating ligands of both species are shown to adopt a planar conformation and in the case of **3** (Figure 4), this is coincident with a crystallographic mirror plane that disorders the hydride and hydroxide substituents over two positions. The dimeric species **4** exhibits a folded structure in which the ligands of each phosphorus center diverge, bringing the two hydrides closer together in space, whilst a slight torsion around the P–O_(μ-O)_ bonds reduce the proximity of the bulky *tert*-butyl groups (Figure 5). The P1–O3 bond of **3** (1.774(3) Å) is significantly longer than both ligand P–O bonds and appreciably longer than the P–O(Ph) bond in the phenol activation product of **B**, [**B**(H)(OPh)] (1.657(2) Å).[26] It is also slightly larger than the sum of the respective covalent radii but well within the sum of the Van der Waals radii.[43, 44] In contrast, the P–O_(μ-O)_ bonds of **4** are much shorter (1.601(2)–1.631(2) Å), consistent with a greater electrostatic interaction between the bridging oxo group and the two phosphorus centers. The ligand P–O and P–N bonds of both species are comparable to those of **2**.

Interestingly, when a solid sample of **3** is heated under a dynamic vacuum at 100 °C for 36 h, the ^31^P NMR spectrum indicates the formation of **4** after redissolving the solid. This suggests that water activation may be reversible (that is, **1** is formed which subsequently reacts with **3** to form **4**). An alternative mechanism involving a condensation reaction between two molecules of **3** is also possible. To probe this further, we decided to investigate the behavior of **2** under related conditions where the possibility of the dimeric imide-bridged system is not possible. Although there is no evidence for reversibility of the oxidative addition product detected in solution, heating solid samples of **2** at 100 °C under a dynamic vacuum does regenerate small amounts of **1**. These observations contrast with those made for [**A**(H)(NH_2_)], which is shown to sublime without decomposition at 40 °C (1 mmHg). The related primary amine and alcohol activation products have, however, been shown to be reversible under forcing conditions.[24]

To conclude, we have shown that the novel phosphorus(III) species **1** is capable of activating the polar E–H bonds of water and ammonia. In the case of water, both of the O–H bonds can be activated sequentially, a transformation which, while postulated in the chemical literature for both transition-metal and main group compounds, has never permitted for the isolation of both the hydride/hydroxide and oxo-bridged products in a main group system. Studies are currently ongoing in our research groups with the goal of converting these interesting stoichiometric reactions into catalytically viable processes capable of transforming abundant and inexpensive small molecules such as H_2_O and NH_3_ into value-added chemicals.

## Supporting Information

As a service to our authors and readers, this journal provides supporting information supplied by the authors. Such materials are peer reviewed and may be re-organized for online delivery, but are not copy-edited or typeset. Technical support issues arising from supporting information (other than missing files) should be addressed to the authors.

miscellaneous_information
