# Supplementary material for: E–H Bond Activation of Ammonia and Water by a Geometrically Constrained Phosphorus(III) Compound
Source: Angew Chem Int Ed Engl. 2015 Sep 22;54(46):13758–63. doi: 10.1002/anie.201506998 (PMC4648037; doi:10.1002/anie.201506998)
Supplement: Supplementary file 1 — miscellaneous_information [file anie0054-13758-sd1.pdf]

Angewandte Chemie
Eine Zeitschrift der Gesellschaft Deutscher Chemiker

Supporting Information

E–H Bond Activation of Ammonia and Water by a Geometrically Constrained Phosphorus(III) Compound

Thomas P. Robinson, Daniel M. De Rosa, Simon Aldridge, and Jose M. Goicoechea**

anie_201506998_sm_miscellaneous_information.pdf

Supporting information

CONTENTS:

1. Experimental section

2. Single crystal X-ray diffraction data

3. Computational analysis

4. NMR spectra

5. ESI-MS spectra

6. IR spectra

7. References

1. Experimental Section

General synthetic methods. All reactions and product manipulations were carried out under an inert atmosphere of argon or dinitrogen using standard Schlenk-line or glovebox techniques (MBraun UNIlab glovebox maintained at < 0.1 ppm H_2O and < 0.1 ppm O_2). H_3[ONO] was synthesized according to a previously reported synthetic procedure.[1] H_2O was obtained from a Millipore Direct-Q 3 water purification device. D_2O (Sigma-Aldrich, 99.9%) was used as received. PCl_3 (Sigma-Aldrich, 99%) was distilled prior to use. NEt_3 (Sigma-Aldrich ≥ 99.5%) was distilled from CaH_2 and degassed by freeze thaw cycles prior to use. NH_3 (BOC, 99.98%) was used as received. Pentane (pent; Sigma-Aldrich, HPLC grade), toluene (Sigma-Aldrich; HPLC grade) and diethyl ether (Sigma-Aldrich; 99.8%) were purified using an MBraun SPS-800 solvent system. Tetrahydrofuran (THF; ≥99.9%, Sigma Aldrich) was distilled over a sodium metal/benzophenone mixture. Pyridine (Py; Alfa Aesar, 99+%) was distilled over CaH_2. C_6D_6 (Sigma Aldrich, 99.6%) was degassed by three freeze-pump-thaw cycles and stored over activated 3Å molecular sieves and used as received. All

dry solvents were stored under argon in gas-tight ampoules. Additionally hexane, pentane, benzene, toluene and THF were stored over activated 3 Å molecular sieves.

Synthesis of P(ONO) (**1**). H_3[ONO] (200 mg, 0.470 mmol) was dissolved in toluene (10 mL) and PCl_3 (41 µL, 0.470 mmol) was added to the solution. Whilst stirring, NEt_3 (228 µL, 1.640 mmol) was added, instantly forming a colourless precipitate. The solution was filtered via cannula and all volatiles were removed *in vacuo*. The resultant residue was washed with cold pentane (3 x 5 mL) after which removal of volatiles *in vacuo* yielded **1** as an off-white solid. Crystals suitable for single crystal X-ray diffraction analysis were grown from a concentrated pentane solution at −30 °C. Yield: 125 mg (59%). CCDC 1415185. Calculated for $C_{28}H_{40}NO_2P$: C 74.14, H 8.89, N 3.09. Observed: C 74.14, H 8.97, N 3.23. ^{1}H NMR (500.30 MHz, C_6D_6, 298 K): δ (ppm) 7.53 (d, $^4J_{H–H}$ = 2.1 Hz, 2H; Ar-C*H*), 7.15 (d, $^4J_{H–H}$ = 2.1 Hz, 2H; Ar-C*H*), 1.39 (s, 18H; *t*Bu), 1.27 (s, 18H; *t*Bu). ^{31}P NMR (162.00 MHz, C_6D_6, 298 K): δ (ppm) 168.6 (s). ^{31}P{^{1}H} NMR (162.00 MHz, C_6D_6, 298 K): δ (ppm) 168.6 (s). ^{13}C{^{1}H} NMR (125.81 MHz, C_6D_6, 298 K): δ (ppm) 145.9 (s; Ar-*C*), 144.0 (d, $^2J_{C–P}$ = 9.7 Hz; Ar-*C*), 138.6 (d, $^2J_{C–P}$ = 1.7 Hz; Ar-*C*), 135.3 (s; Ar-*C*), 119.6 (s; Ar-*C*H), 114.3 (d, $^3J_{C–P}$ = 7.1 Hz; Ar-*C*H), 34.8 (s; *t*Bu-*C*), 34.8 (s; *t*Bu-*C*), 31.7 (s; *t*Bu-*C*H_3), 29.6 (s; *t*Bu-*C*H_3). EI-MS: calc. for $C_{28}H_{40}NO_2P$ (M^+) 453.2870, found 453.2788, calc. for $C_{27}H_{37}NO_2P$ 438.2635, found 438.2520.

Synthesis of P(ONO)(NH_2)(H) (**2**). **1** (200 mg, 0.441 mmol) was dissolved in toluene (10 mL) in an air-tight ampoule. The solution was freeze-pump-thaw degassed three times and left under vacuum. The ampoule was placed under one atmosphere of NH_3 (g) and the solution was stirred for 1 hour. All volatiles were removed *in vacuo* and the resultant residue was extracted into toluene and filtered via cannula. The solution was concentrated and

crystals of **2** suitable for single crystal X-ray diffraction analysis were grown at −30 °C. Yield: 178 mg (86%). CCDC 1415186. Calculated for $C_{28}H_{42}N_2O_2P$: C 71.61, H 9.01, N 5.97. Observed: C 71.50, H 9.29, N 5.83. ^{1}H NMR (500.03 MHz, C_6D_6, 298 K): δ (ppm) 8.46 (d, $^1J_{H–P}$ = 818.9 Hz, 1H; P*H*), 7.74 (s br, 2H; Ar-C*H*), 7.15 (d, $^4J_{H–H}$ = 1.8 Hz, 2H, Ar-C*H*), 2.16 (d, $^2J_{H–P}$ = 11.4 Hz, 2H; NH_2), 1.51 (s, 18H; *t*Bu), 1.43 (s, 18H; *t*Bu). ^{1}H{^{31}P} NMR (500.03 MHz, C_6D_6, 298 K): δ (ppm) 8.46 (s; P*H*), 7.74 (s br; Ar-C*H*), 7.15 (d, $^4J_{H–H}$ = 1.8 Hz, Ar-C*H*), 2.16 (s; NH_2), 1.51 (s; *t*Bu), 1.43 (s; *t*Bu). ^{31}P NMR (161.99 MHz, C_6D_6, 298 K): δ (ppm) −46.1 (dt, $^1J_{P–H}$ = 818.9 Hz, $^2J_{P–H}$ = 11.4 Hz). ^{31}P{^{1}H} NMR (161.99 MHz, C_6D_6, 298 K): δ (ppm) −46.1 (s). ^{13}C{^{1}H} NMR (100.63 MHz, C_6D_6, 298 K): δ (ppm) 143.5 (d, $^4J_{C–P}$ = 3.0 Hz; Ar-*C*), 142.3 (Ar-*C*), 131.8 (d, $^2J_{C–P}$ = 4.6 Hz; Ar-C), 129.3 (d, $^2J_{C–P}$ = 20.5 Hz; Ar-*C*), 115.6 (Ar-*C*H), 106.8 (d, $^3J_{C–P}$ = 13.4 Hz; Ar-*C*H), 35.0 (*t*Bu-*C*), 34.6 (*t*Bu-*C*), 32.0 (*t*Bu-*C*H_3) 29.8 (*t*Bu-*C*H_3). EI-MS: calc. for $C_{28}H_{43}N_2O_2P$ (M^+) 470.3062, found 470.3057, calc. for $C_{28}H_{40}NO_2P$ 453.2870, found 453.2737, calc. for C27H37NO2P 438.2635, found 438.2417.

Synthesis of P(ONO)(OH)(H) (**3**). To a diethyl ether solution of **1** (200 mg, 0.441 mmol, 10 mL) was added H_2O (16 μL, 0.882 mmol). The resultant solution was stirred for 2 hours after which all volatiles were removed *in vacuo*. The resulting residue was extracted into toluene and filtered via cannula. All volatiles were removed *in vacuo* yielding **3** as a colourless solid. Crystals suitable for single crystal X-ray diffraction analysis were grown from a concentrated toluene solution at −30 °C. Yield: 127 mg (61%). CCDC 1415187. Calculated for $C_{28}H_{42}NO_3P$: C 71.31, H 8.98, N 2.97. Observed: C 71.84, H 9.09, N 2.92. ^{1}H NMR (500.30 MHz, C_6D_6, 298 K): δ (ppm) 8.22 (d, $^1J_{H–P}$ = 880.6 Hz, 1H; P*H*), 7.79 (s br, 2H; Ar-C*H*), 7.19 (d, $^4J_{H–H}$ = 1.6 Hz; Ar-C*H*), 3.87 (s br, 1H, O*H*), 1.52 (s, 18H; *t*Bu), 1.43 (s, 18H; *t*Bu). ^{1}H{^{31}P} NMR (500.30 MHz, C_6D_6, 298 K): δ (ppm) 8.22 (s; P*H*), 7.79 (s br, 2H; Ar-C*H*),

7.19 (d, $^4J_{H–H}$ = 1.6 Hz; Ar-C*H*), 3.87 (s br, 1H, O*H*), 1.52 (s, 18H; *t*Bu), 1.43 (s, 18H; *t*Bu). ^{31}P NMR (202.37 MHz, C_6D_6, 298 K): δ (ppm) –36.9 (d, $^1J_{P–H}$ = 880.6 Hz). ^{31}P{^{1}H} NMR (202.37 MHz, C_6D_6, 298 K): δ (ppm) –36.9 (s). ^{13}C{^{1}H} NMR (125.81 MHz, C_6D_6, 298 K): δ (ppm) 142.8 (s; Ar-*C*), 142.6 (d, $^2J_{C–P}$ = 4.7 Hz; Ar-*C*), 132.5 (d, $^2J_{C–P}$ = 5.1 Hz; Ar-*C*), 128.6 (s; Ar-*C*), 116.1 (s; Ar-*C*H), 106.9 (d, $^3J_{C–P}$ = 14.3 Hz; Ar-*C*H), 35.0 (s; *t*Bu-*C*), 34.7 (s; *t*Bu-*C*), 32.0 (s; *t*Bu-*C*H$_3$), 29.8 (s; *t*Bu-*C*H$_3$). EI-MS: calc. for $C_{28}H_{42}NO_3P$ (M^+) 471.2902, found 471.2901, calc. for $C_{28}H_{40}NO_2P$ 453.2870, found 453.2654, calc. for $C_{27}H_{37}NO_2P$ 438.2635, found 438.2398.

Synthesis of [P(ONO)(H)]$_2$μ-O (**4**). **1** (50 mg, 0.110 mmol) and **3** (52 mg, 0.110 mmol) were dissolved in toluene (5 mL) and the solution was stirred for 12 hours at 70 °C. The solution was filtered via cannula and all volatiles were removed *in vacuo*, giving **4** as a colourless solid. Crystals suitable for single crystal X-ray diffraction analysis were grown from a concentrated pyridine solution at −30 °C. Yield: 76 mg (75%). CCDC 1415188. Calculated for $C_{56}H_{82}N_2O_5P_2 \cdot 2(C_5H_5N)$: C 73.17, H 8.56, N 5.17. Observed: C 71.88, H 8.46, N 5.12. ^{1}H NMR (400.16 MHz, C_6D_6, 298 K): δ (ppm) 8.43 (m, $^1J_{H–P}$ = 912.5 Hz, $^3J_{H–P}$ = 0.7 Hz, $^2J_{P–P}$ = -30.2 Hz, 2H; P*H*)*, 7.66 (d, $^4J_{H–H}$ = 1.4 Hz, 2H; Ar-C*H*), 7.11 (d, $^4J_{H–H}$ = 1.4 Hz, 2H; Ar-C*H*), 1.47 (s br, 18H; *t*Bu), 1.37 (s, 18H; *t*Bu). ^{1}H{^{31}P} NMR (500.30 MHz, C_6D_6, 298 K): δ (ppm) 8.43 (m; P*H*)** 7.66 (d, $^4J_{H–H}$ = 1.4 Hz; Ar-C*H*), 7.11 (d, $^4J_{H–H}$ = 1.4 Hz; Ar-C*H*), 1.47 (s br; *t*Bu), 1.37 (s; *t*Bu). ^{31}P NMR (161.99 MHz, C_6D_6, 298 K): δ (ppm) −44.0 (m, $^2J_{P–P}$ = –30.2 Hz, $^1J_{P–H}$ = 912.5 Hz, $^3J_{P–H}$ = 0.7 Hz)*. ^{31}P{^{1}H} NMR (161.99 MHz, C_6D_6, 298 K): δ (ppm) −44.0 (s). ^{13}C{^{1}H} NMR (100.63 MHz, C_6D_6, 298 K): δ (ppm) 143.4 (Ar-*C*), 140.8 (m; Ar-*C*), 133.2 (Ar-*C*), 128.6 (Ar-*C*), 115.9 (Ar-*C*H), 106.6 (m; Ar-*C*H), 35.0 (*t*Bu-*C*), 34.7 (*t*Bu-*C*), 31.9 (*t*Bu-*C*H), 29.9 (*t*Bu-*C*H). EI-MS: calc. for $C_{56}H_{80}N_2O_5P$ (M^+) 924.5699, found

924.1963, calc. for $C_{28}H_{42}NO_3P$ 471.2902, found 471.0569, calc. for $C_{28}H_{40}NO_2P$ 453.2870, found 453.0557, calc. for $C_{27}H_{37}NO_2P$ 438.2635, found 438.0374.

* Coupling constants obtained from iterated simulations performed using the gNMR software.

** Full decoupling of resonance was not possible on account of the very large $^1J_{H-P}$ coupling constant.

Reversibility of NH_3 activation

Samples of **2** were added to an ampoule and heated at 100 ^{o}C under dynamic vacuum for 36 h. The resulting residue was analysed by ^{31}P NMR showing small amounts of conversion to the starting material **1** in addition to a resonance corresponding to **4**, presumably formed through the reaction of **1** with small amounts of water.

Reversibility of H_2O activation

Samples of **3** were added to an ampoule and heated at 100 ^{o}C under dynamic vacuum for 36 h. The resulting residue was analysed by ^{31}P NMR showing small amounts of conversion to **4**. This may occur via initial formation of **1** and subsequent reaction with **3**, or via a condensation reaction between two molecules of **3**.

D_2O exchange reactions with 3

Two samples of **3** (30 mg, 0.06 mmol) were dissolved in THF (0.5 mL) and D_2O (1.1 μL, 0.06 mmol) was added to each sample. One sample was allowed to react at room temperature for 2 hours and the second at 70 ^{o}C for two hours. All volatiles were removed from each sample *in vacuo* and the resulting solids were analysed by ^{2}D NMR spectroscopy. The reaction at room temperature showed significant exchange of the hydroxyl proton of **3** but

negligible exchange of the phosphorus bound hydride. The reaction that was heated at 70 °C showed comparable exchange at both sites.

<u>*Reaction at room temperature:*</u> ^{2}D NMR (76.74 MHz, C_6D_6, 298 K): δ (ppm) 8.75 (weak d, $^{1}J_{D-P}$ = 133 Hz; P*D*), 8.31 (br s, O*D*).

<u>*Reaction at 70 °C:*</u> ^{2}D NMR (76.74 MHz, C_6D_6, 298 K): δ (ppm) 8.75 (strong d, $^{1}J_{D-P}$ = 133 Hz; P*D*), 8.31 (br s, O*D*).

Single crystal X-ray structure determination: Single-crystal X-ray diffraction data were collected using an Oxford Diffraction Supernova dual-source diffractometer equipped with a 135 mm Atlas CCD area detector. Crystals were selected under Paratone-N oil, mounted on micromount loops and quench-cooled using an Oxford Cryosystems open flow S.I.7 N_2 cooling device. Data were collected at 150 K using mirror monochromated Cu Kα radiation (λ = 1.5418 Å) and processed using the CrysAlisPro package, including unit cell parameter refinement and inter-frame scaling (which was carried out using SCALE3 ABSPACK within CrysAlisPro).[2] Equivalent reflections were merged and diffraction patterns processed with the CrysAlisPro suite. Structures were subsequently solved using direct methods and refined on F^2 using the SHELXL 2014-3 package.[3]

Additional characterization techniques: ^{1}H, ^{2}D, ^{13}C, and ^{31}P spectra were acquired at either 500.3, 125.8 and 202.4 MHz, respectively, on a Bruker AVIII 500 MHz NMR Spectrometer or 400.2, 100.6 and 162.0 MHz, respectively, on a Bruker AVIII HD nanobay 400 MHz NMR Spectrometer. ^{1}H and ^{13}C NMR spectra were referenced to the most downfield solvent resonance (^{1}H NMR C_6D_6: δ = 7.16 ppm; ^{13}C NMR C_6D_6: δ = 128.06 ppm). ^{2}H NMR spectra were referenced to the deuterium resonance of C_6D_6 collected prior to collection of each

sample. ^{31}P NMR spectra were externally referenced to an 85% solution of H_3PO_4 in H_2O (δ = 0 ppm).

Electron Impact mass spectra were obtained on a neat sample using a Waters GCT Time of Flight Mass Spectrometer with a temperature programmed solids probe inlet. Samples were ionised using an electron impact ionisation technique with 70 eV electron energy.

Infrared spectra were collected on a Thermo Scientific Nicolet iS5 FT-IR spectrometer in absorbance mode. Samples were prepared as KBr disks.

2. Single crystal X–ray diffraction data

Table S1. Selected X–ray data collection and refinement parameters for **1**, **2**, **3** and **4**·2py.

	**1**	**2**	**3**	**4**·2py
Formula	$C_{28}H_{40}NO_2P$	$C_{28}H_{43}N_2O_2P$	$C_{28}H_{42}NO_3P$	$C_{66}H_{92}N_4O_5P_2$
Fw [g mol^{-1}]	453.58	470.61	471.59	1083.37
crystal system	triclinic	triclinic	orthorhombic	monoclinic
space group	*P*–1	*P*–1	*Ibam*	*C*2
a (Å)	10.6988(6)	5.9091(2)	19.6698(5)	26.3236(6)
b (Å)	11.4386(4)	16.5724(4)	29.5497(7)	17.2027(3)
c (Å)	12.7832(5)	28.1697(8)	9.5581(3)	29.3386(6)
α (°)	96.419(3)	89.600(2)	90	90
β (°)	100.990(4)	88.441(2)	90	108.559(2)
γ (°)	117.260(5)	89.293(2)	90	90
V (Å^3)	1329.13(12)	2757.31(14)	5555.5(3)	12594.7(5)
Z	2	4	8	8
radiation, λ (Å)	Cu K_α (1.54178)			
T (K)	150(2)			
ρ_{calc} (g cm^{-3})	1.133	1.134	1.128	1.143
μ (mm^{-1})	1.084	1.071	1.081	1.014
reflections collected	22883	27112	24560	68681
independent reflections	5502	9885	2758	23195
parameters	317	651	234	1413
R(int)	0.0240	0.0229	0.0238	0.0373
R1/wR2,[a] I ≥ 2σI (%)	4.00/10.39	6.31/15.77	5.00/12.36	3.84/9.69
R1/wR2,[a] all data (%)	4.43/10.88	6.62/15.90	5.12/12.44	4.44/10.10
GOF	1.039	1.189	1.086	1.033

[a] $R1 = [\Sigma||F_o| - |F_c||]/\Sigma|F_o|$; $wR2 = \{[\Sigma w[(F_o)^2 - (F_c)^2]^2]/[\Sigma w(F_o^2)^2]\}^{1/2}$; $w = [\sigma^2(F_o)^2 + (AP)^2 + BP]^{-1}$, where $P = [(F_o)^2 + 2(F_c)^2]/3$ and the A and B values are 0.0551 and 0.53 for **1**, 0.0362 and 4.44 for **2**, 0.0564 and 6.34 for **3** and 0.0628 and 0.92 for **4**·2py.

Table S1. A comparison of the bond metrics of the two independent molecules of **2** in the crystal lattice.

**Bond Lengths (Å) and Angles (°) for molecule I**		**Bond Lengths (Å) and Angles (°) for molecule II**	
P1–N1	1.700(2)	P101–N101	1.702(2)
P1–O1	1.718(2)	P101–O101	1.712(2)
P1–O2	1.710(2)	P101–O102	1.715(2)
O1–P1–O2	176.67(11)	O101–P101–O102	176.67(11)
N1–P1–O1	88.64(11)	N101–P101–O101	88.33(11)
N1–P1–O2	88.36(11)	N101–P101–O102	88.64(10)

Table S2. A comparison of the bond metrics of the two independent molecules of **4** in the crystal lattice.

Bond Lengths (Å) and Angles (°) for molecule **I**		Bond Lengths (Å) and Angles (°) for molecule **II**	
P1–N1	1.710(2)	P11–N11	1.706(2)
P1–O1	1.693(2)	P11–O11	1.6764(18)
P1–O2	1.680(2)	P11–O12	1.6844(17)
P1–O3	1.628(2)	P11–O13	1.6023(19)
P1–H1	1.30(3)	P11–H11	1.30(3)
P2–N2	1.707(2)	P12–N12	1.710(2)
P2–O3	1.601(2)	P12–O13	1.6310(19)
P2–O4	1.680(2)	P12–O14	1.6788(17)
P2–O5	1.692(2)	P12–O15	1.6754(17)
P2–H2	1.24(3)	P12–H12	1.22(3)

P1–O3–P2	134.8(1)	P11–O3–P12	135.12(12)
O1–P1–O2	166.0(1)	O11–P11–O12	161.58(10)
O1–P1–O3	97.9(1)	O11–P11–O13	98.45(10)
O1–P1–H1	87.4(12)	O11–P11–H11	85.8(14)
O2–P1–O3	96.1(1)	O12–P11–O13	99.93(9)
O2–P1–H1	88.7(12)	O12–P11–H11	87.8(14)
O3–P1–H1	102.5(14)	O13–P11–H11	106.6(15)
N1–P1–O1	89.0(1)	N11–P11–O11	88.55(10)
N1–P1–O2	89.0(1)	N11–P11–O12	88.37(9)
N1–P1–O3	102.2(1)	N11–P11–O13	103.68(10)
N1–P1–H1	155.3(14)	N11–P11–H11	149.7(15)
O4–P2–O5	163.7(1)	O14–P12–O15	164.41(10)
O4–P2–O3	96.8(1)	O14–P12–O13	98.32(9)
O4–P2–H2	88.6(16)	O14–P12–H12	85.8(14)
O5–P2–O3	99.4(1)	O15–P12–O13	97.24(9)
O5–P2–H2	84.7(16)	O15–P12–H12	89.4(14)
O3–P2–H2	110.2(17)	O13–P12–H12	103.9(15)
N2–P2–O3	106.0(1)	N12–P12–O13	101.81(10)
N2–P2–O4	88.3(1)	N12–P12–O14	88.97(9)
N2–P2–O5	88.4(1)	N12–P12–O15	88.88(9)
N2–P2–H2	143.7(17)	N12–P12–H12	154.2(15)

3. Computational details

All geometry optimizations were performed using the Amsterdam Density Functional package (ADF2013.01).[4] An TZP Slater-type basis set of triple-ζ quality, extended with one polarization function, was used to describe all phosphorus, nitrogen and oxygen atoms while a DZP basis set was used for all remaining atoms. Geometry optimizations were performed using the hybrid Becke three-parameter functional with Lee-Yang-Parr correlation (B3LYP).[5–7] All structures were optimized using the gradient algorithm of Versluis and Ziegler.[8]

Coordinates [Å] for the optimized geometry of 1 (C_s symmetry)

**Atom**	**x**	**y**	**z**
1. P	0.000000000	0.000000000	0.000000000
2. N	0.000000000	1.792012153	0.000000000
3. O	–1.456015656	–0.059338466	–0.840631031
4. O	1.318031819	–0.051240311	–1.075784680
5. C	–1.970464487	1.199807878	–1.163655131
6. C	–3.152906983	1.427366173	–1.892452357
7. C	–3.508759524	2.781478165	–2.073426438
8. H	–4.409164274	2.999858869	–2.627823860
9. C	–2.753142399	3.863128965	–1.568749448
10. C	–1.584360540	3.581240943	–0.838021649
11. H	–0.981703845	4.375326071	–0.425576853
12. C	–1.193182147	2.250598407	–0.653779337
13. C	–3.997708025	0.261254984	–2.458182318
14. C	–5.242015468	0.772510968	–3.235756335

15. H	–4.955178180	1.390467041	–4.095797103
16. H	–5.916885228	1.344465627	–2.586727160
17. H	–5.800532036	–0.091262620	–3.617991205
18. C	–4.498083001	–0.630972705	–1.279508534
19. H	–3.660598161	–1.071825038	–0.731097434
20. H	–5.127011203	–1.442965419	–1.667790344
21. H	–5.093169728	–0.030077708	–0.579523530
22. C	–3.122928375	–0.574633227	–3.443422886
23. H	–2.259313253	–1.013040555	–2.935223856
24. H	–2.757959312	0.064760165	–4.257880978
25. H	–3.721513866	–1.387518107	–3.875719589
26. C	–3.207292493	5.315787326	–1.865426345
27. C	–3.099660594	5.568421701	–3.401678455
28. H	–3.378145957	6.604662312	–3.635831370
29. H	–3.761325421	4.898974441	–3.963446267
30. H	–2.071675594	5.392032709	–3.741426125
31. C	–2.319627380	6.364003656	–1.139786829
32. H	–2.344241399	6.225075450	–0.051368067
33. H	–2.697919632	7.370684166	–1.359245336
34. H	–1.280956706	6.319026239	–1.487531472
35. C	–4.678968674	5.518821879	–1.394219588
36. H	–4.769604936	5.298500660	–0.323199691
37. H	–5.372116009	4.866181288	–1.936533416
38. H	–4.988972099	6.557597230	–1.568455693
39. C	1.916123248	1.196948560	–1.251162957

40. C	3.098457082	1.431252424	–1.971091834
41. C	3.568647112	2.768836754	–1.972054086
42. H	4.474769097	2.983000920	–2.515463681
43. C	2.925494467	3.822025991	–1.295816449
44. C	1.739800803	3.534483344	–0.583778227
45. H	1.226053971	4.304434861	–0.024968889
46. C	1.233745481	2.236848456	–0.588413049
47. C	3.832502789	0.296435727	–2.723908867
48. C	2.866418017	–0.319005096	–3.783825258
49. H	1.979743449	–0.751067093	–3.310696627
50. H	3.382434255	–1.109806077	–4.344728758
51. H	2.538927420	0.456272146	–4.488857870
52. C	4.288472534	–0.790308218	–1.701152727
53. H	3.432199807	–1.237534009	–1.188723988
54. H	4.949751882	–0.343481720	–0.947234802
55. H	4.837526190	–1.585832042	–2.222532334
56. C	5.093822517	0.815737969	–3.467942917
57. H	5.566765625	–0.024870286	–3.991438426
58. H	5.832201596	1.231796120	–2.771344277
59. H	4.836793014	1.573221517	–4.218827103
60. C	3.464791812	5.275292531	–1.313286684
61. C	2.384931993	6.212444556	–1.938813099
62. H	1.469687080	6.227438900	–1.336551873
63. H	2.123098459	5.868763310	–2.947437578
64. H	2.766476844	7.240203711	–2.001728329

65. C	3.780958996	5.726165153	0.145868711
66. H	4.522835573	5.054597754	0.596728075
67. H	2.884006056	5.711687866	0.776279351
68. H	4.181922223	6.748437477	0.147603927
69. C	4.763451524	5.412393942	–2.154548968
70. H	5.573682180	4.794202617	–1.748411454
71. H	5.097617079	6.457634188	–2.131607177
72. H	4.592518115	5.139559277	–3.203477508

TOTAL BONDING ENERGY: –45331.72 kJ mol^{-1}

Coordinates [Å] for the optimized geometry of 1 (C_{2v} symmetry)

1. P	0.000000000	0.000000000	0.000000000
2. N	0.000000000	1.772007400	0.000000000
3. O	–1.793441226	0.150940991	–0.028853709
4. O	1.793431610	0.151800799	0.000000000
5. C	–1.262431087	2.384577350	–0.008601897
6. C	–1.608968508	3.753637400	0.007747170
7. H	–0.855875187	4.514917021	0.022934295
8. C	–2.955188718	4.131594513	0.002237706
9. C	–3.951261653	3.120477986	–0.030509672
10. H	–4.987045011	3.426000828	–0.040151343
11. C	–3.660458844	1.751519275	–0.052547369
12. C	–2.282279183	1.399411550	–0.031592880
13. C	1.262503514	2.385153295	–0.002798014

14. C	2.282091832	1.399718310	–0.007877235
15. C	3.660409173	1.751165699	–0.021943559
16. H	4.987275440	3.425471404	–0.034004840
17. C	3.951531978	3.120255986	–0.024612315
18. C	2.955661982	4.131867096	–0.018033500
19. H	0.855616604	4.514942121	0.003602706
20. C	1.609462318	3.754337743	–0.004932488
21. C	4.757902639	0.662199706	–0.033438108
22. C	6.183223765	1.279261068	–0.066608976
23. H	6.920841895	0.466954704	–0.080587031
24. H	6.340654271	1.886763362	–0.966903803
25. H	6.379699669	1.890178696	0.823283224
26. C	4.633557076	–0.204993418	1.258560890
27. H	4.743091760	0.428194727	2.149147249
28. H	5.421894200	–0.969324661	1.271594927
29. H	3.662422636	–0.705750563	1.305704778
30. C	4.586662928	–0.228146888	–1.304556879
31. H	4.645192023	0.390038466	–2.210409704
32. H	5.385344145	–0.981021921	–1.341054729
33. H	3.621950795	–0.743924006	–1.297961978
34. C	3.391484454	5.619382293	–0.017730558
35. C	4.267487441	5.901245514	–1.277868416
36. H	4.557520294	6.959975479	–1.305967195
37. H	5.183750457	5.299734165	–1.276708288
38. H	3.705541627	5.663827805	–2.191373739

39. C	2.177035225	6.588056298	–0.051591859
40. H	1.568081262	6.437961965	–0.951544941
41. H	2.542968776	7.622463654	–0.066943590
42. H	1.546171630	6.474629159	0.838384530
43. C	4.208972405	5.911000944	1.279461748
44. H	5.123785240	5.308237759	1.322232662
45. H	4.496285344	6.970300730	1.315218773
46. H	3.606155010	5.677414296	2.166942512
47. C	–4.758259349	0.663569754	–0.099632328
48. C	–4.640309420	–0.244896500	1.163593490
49. H	–5.436714550	–1.001137019	1.153957878
50. H	–3.674301122	–0.756177287	1.193052634
51. H	–4.742196898	0.360561531	2.073871716
52. C	–6.183914019	1.280233233	–0.117877947
53. H	–6.381915802	1.866910290	0.788061463
54. H	–6.920698886	0.467813135	–0.155646085
55. H	–6.340603497	1.911796699	–1.000979772
56. C	–4.582274339	–0.184981174	–1.398089348
57. H	–3.612834894	-0.690980153	–1.411129694
58. H	–5.373297776	-0.944430481	–1.456158178
59. H	–4.650289385	0.461156344	–2.283348566
60. C	–3.390158193	5.619025864	0.034995461
61. C	–2.175051029	6.585820523	0.062788166
62. H	–2.539036120	7.620680889	0.087841567
63. H	–1.557509891	6.430249236	0.956040702

64. H	–1.552744312	6.475364391	–0.833653887
65. C	–4.233797878	5.937900318	–1.237792017
66. H	–3.649368336	5.727063734	–2.142932432
67. H	–4.524652543	6.996627707	–1.242847583
68. H	–5.148501565	5.335089227	–1.276962258
69. C	–4.240612440	5.874447538	1.318412544
70. H	–3.658889411	5.624391096	2.214536702
71. H	–4.536107732	6.930411573	1.371091608
72. H	–5.151990651	5.265257985	1.324431643

TOTAL BONDING ENERGY: –45327.78 kJ mol^{-1}

4. NMR Spectra

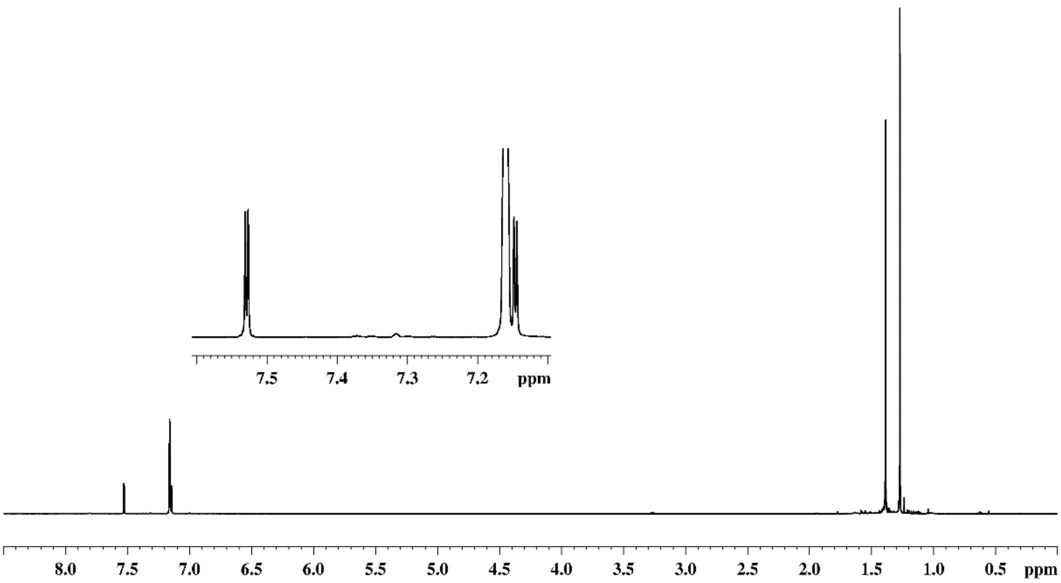

Figure S1. Room temperature ^{1}H NMR spectrum of **1** (C_6D_6).

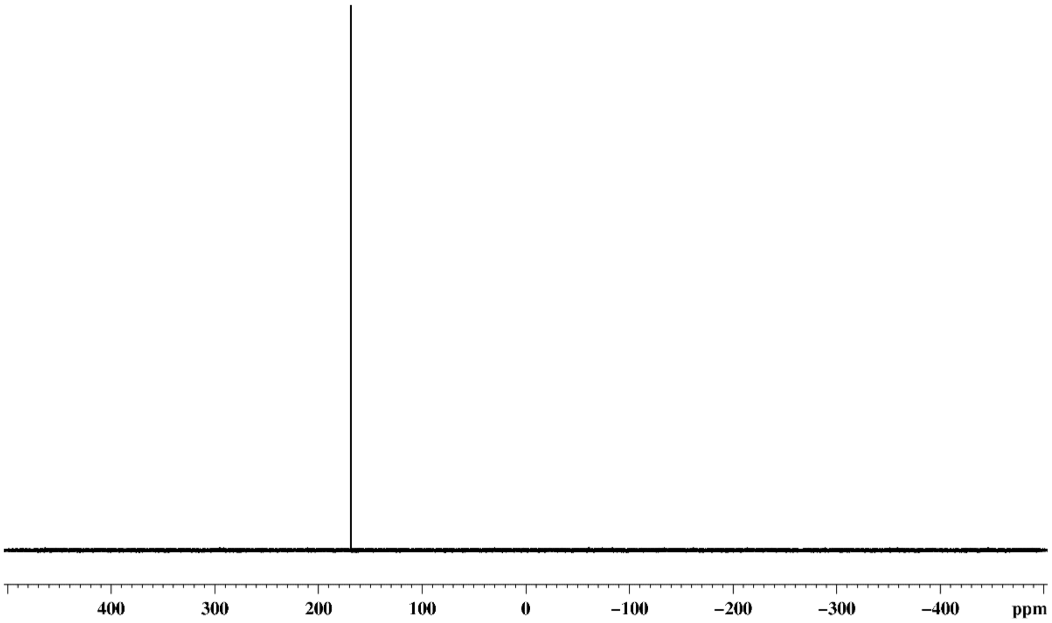

Figure S2. Room temperature ^{31}P NMR Spectrum of **1** (C_6D_6).

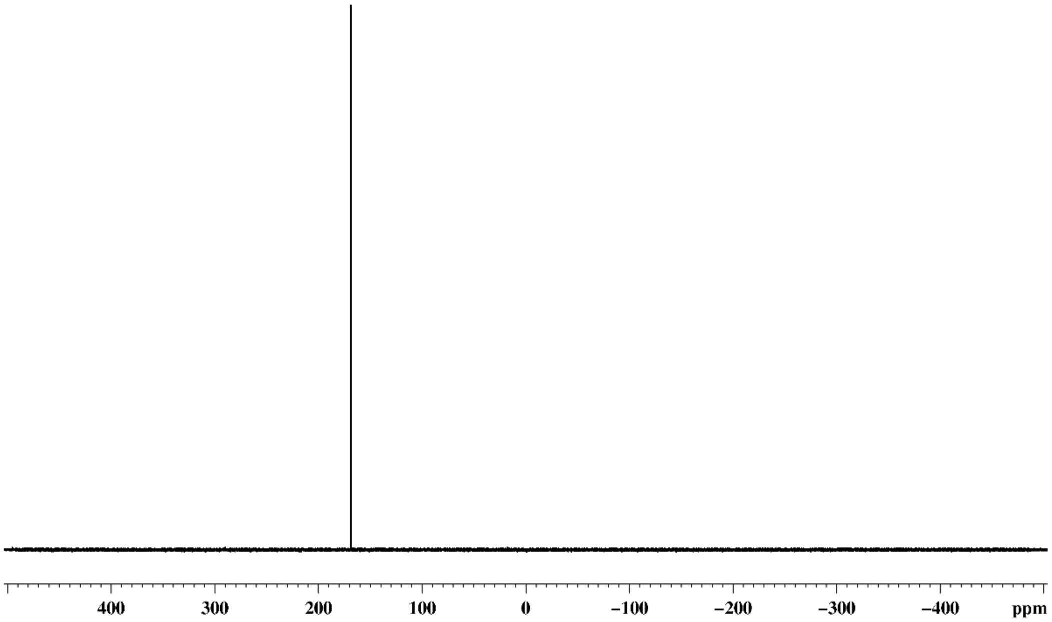

Figure S3. Room temperature $^{31}P\{^{1}H\}$ NMR spectrum of **1** (C_6D_6).

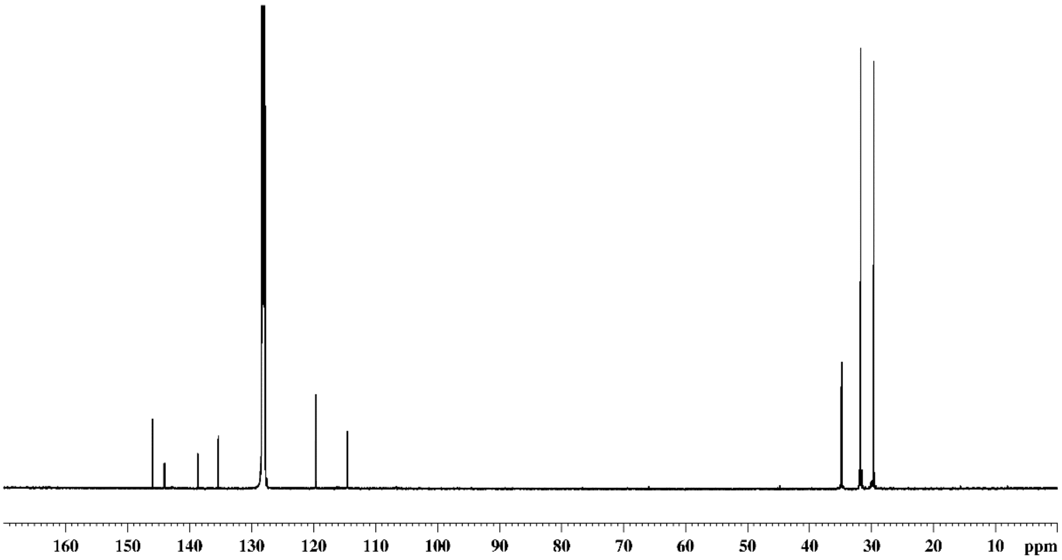

Figure S4. Room temperature $^{13}C\{^{1}H\}$ NMR spectrum of **1** (C_6D_6).

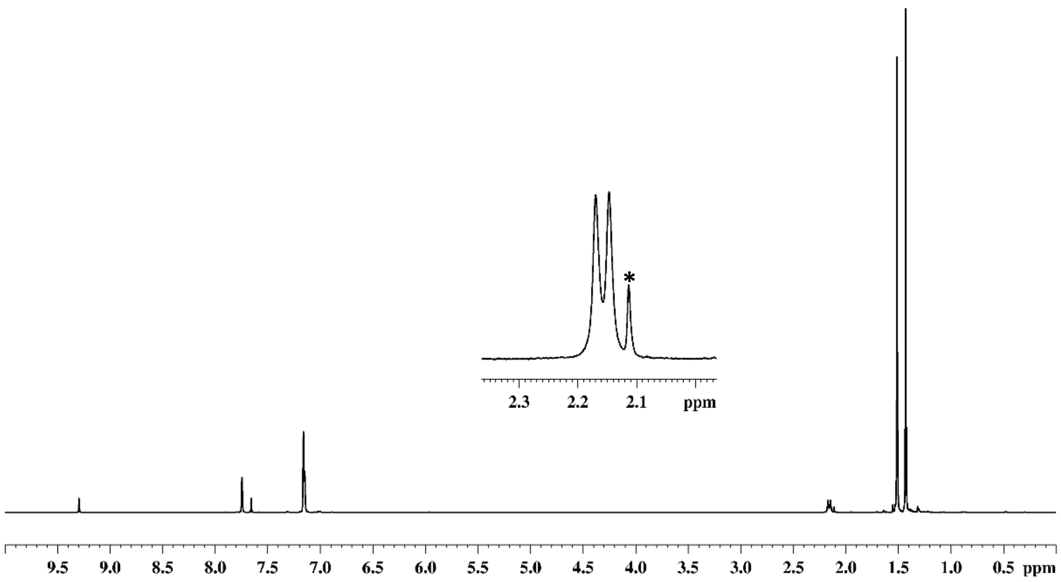

Figure S5. Room temperature ^{1}H NMR spectrum of **2** (C_6D_6). * Denotes residual toluene

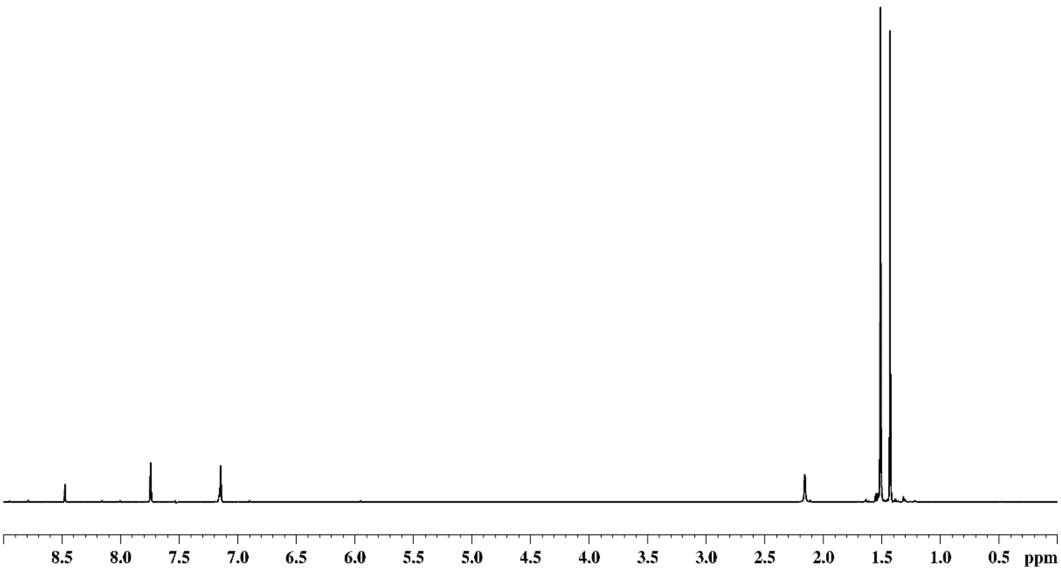

Figure S6. Room temperature $^{1}H\{^{31}P\}$ NMR Spectrum of **2** (C_6D_6).

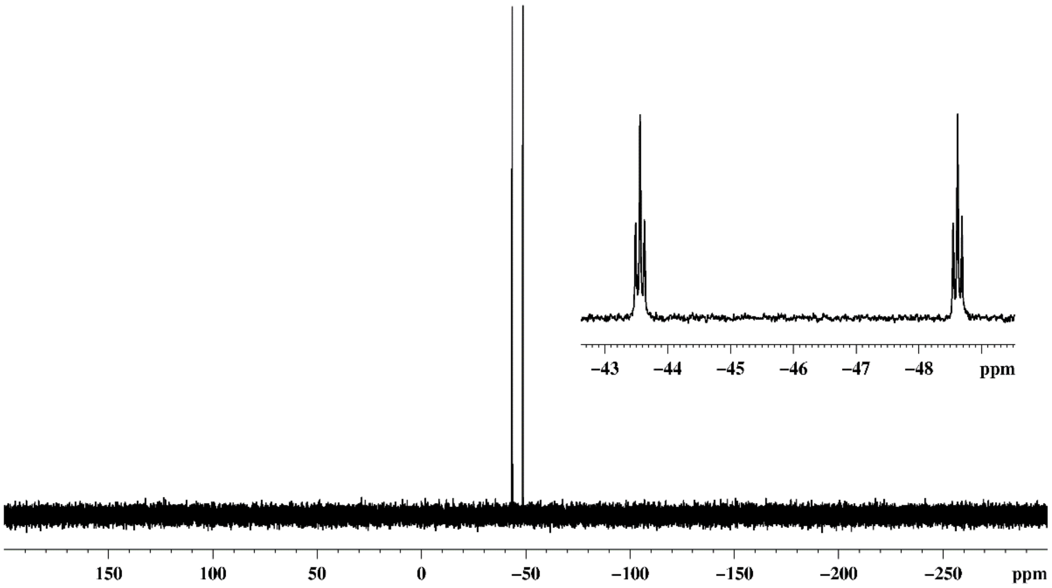

Figure S7. Room temperature ^{31}P NMR spectrum of **2** (C_6D_6).

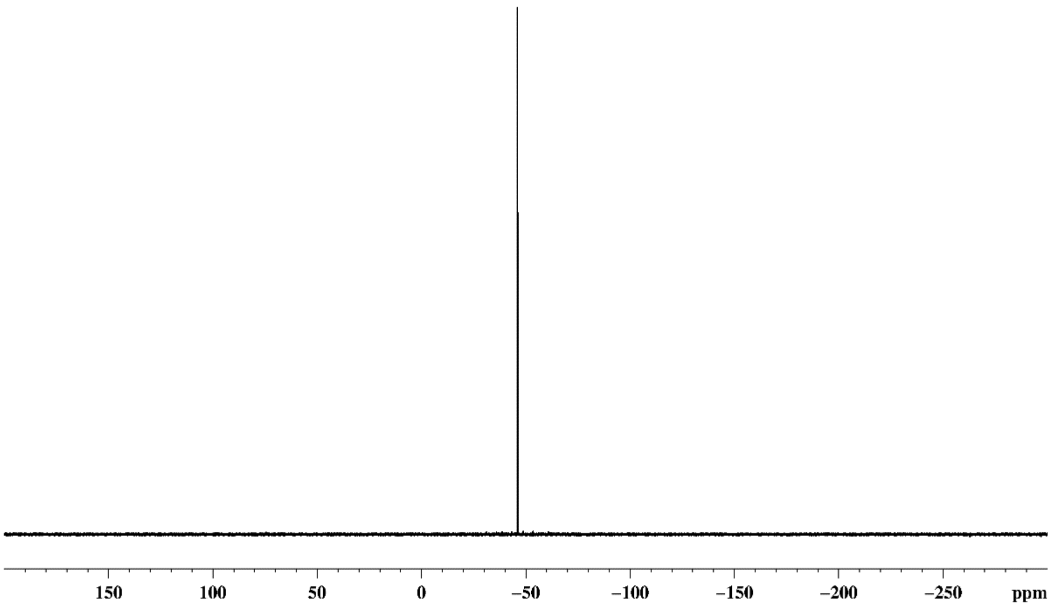

Figure S8. Room temperature ^{31}P{^{1}H} NMR spectrum of **2** (C_6D_6).

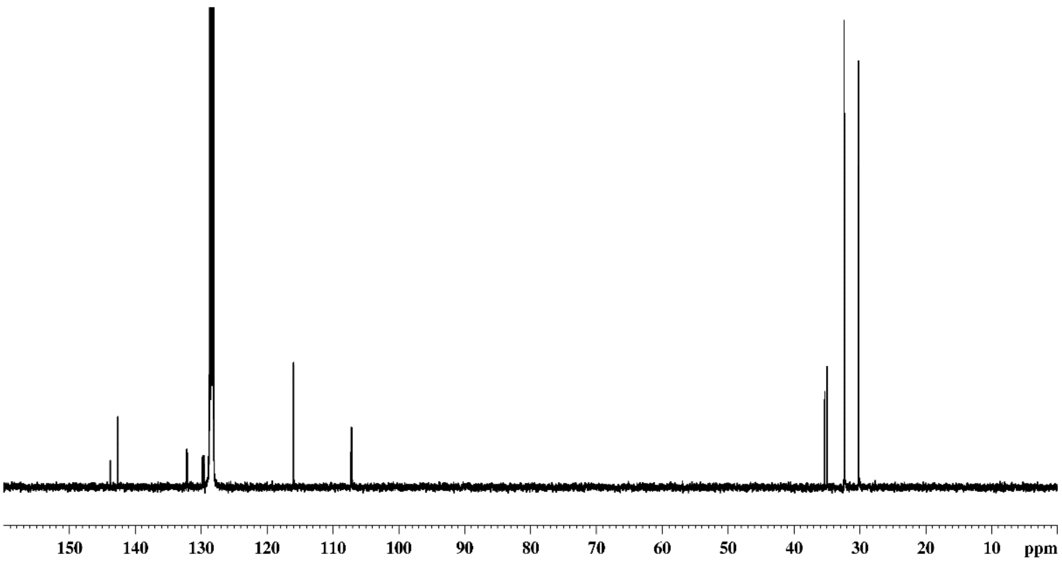

Figure S9. Room temperature $^{13}C\{^1H\}$ NMR spectrum of **2** (C_6D_6).

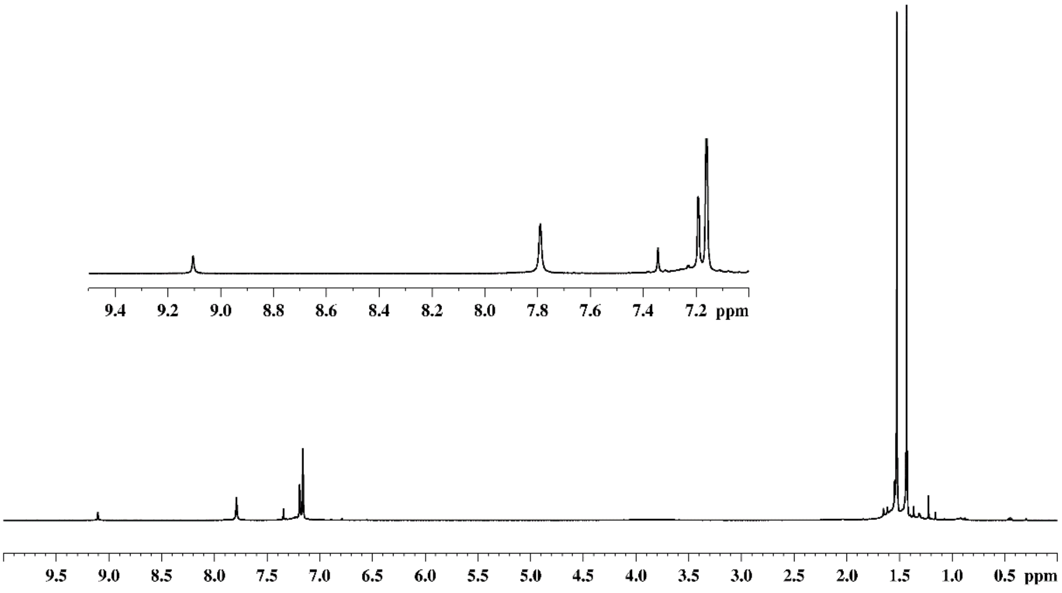

Figure S10. Room temperature ^1H NMR spectrum of **3** (C_6D_6).

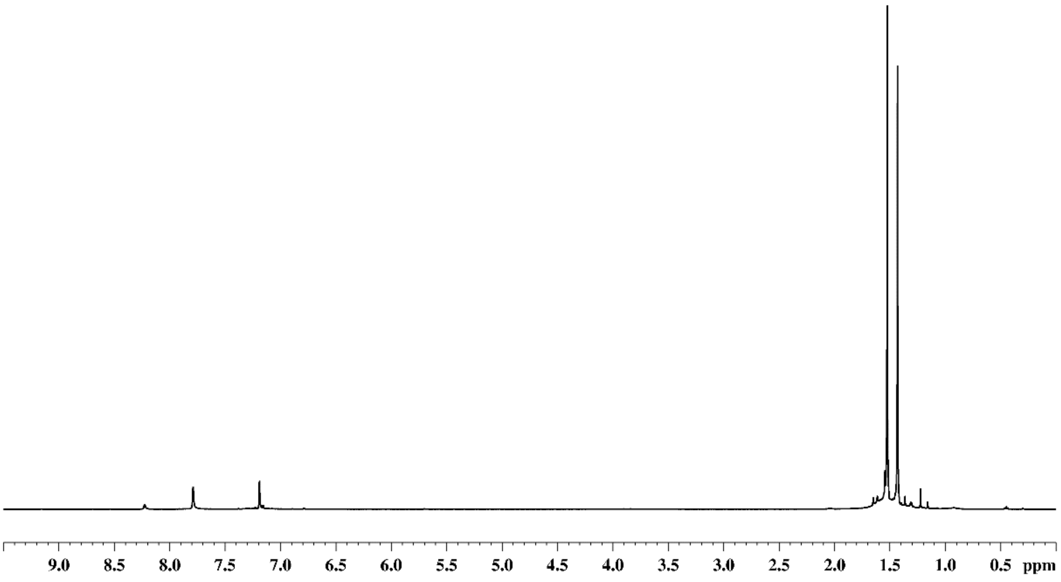

Figure S11. Room temperature $^{1}H\{^{31}P\}$ NMR spectrum of **3** (C_6D_6).

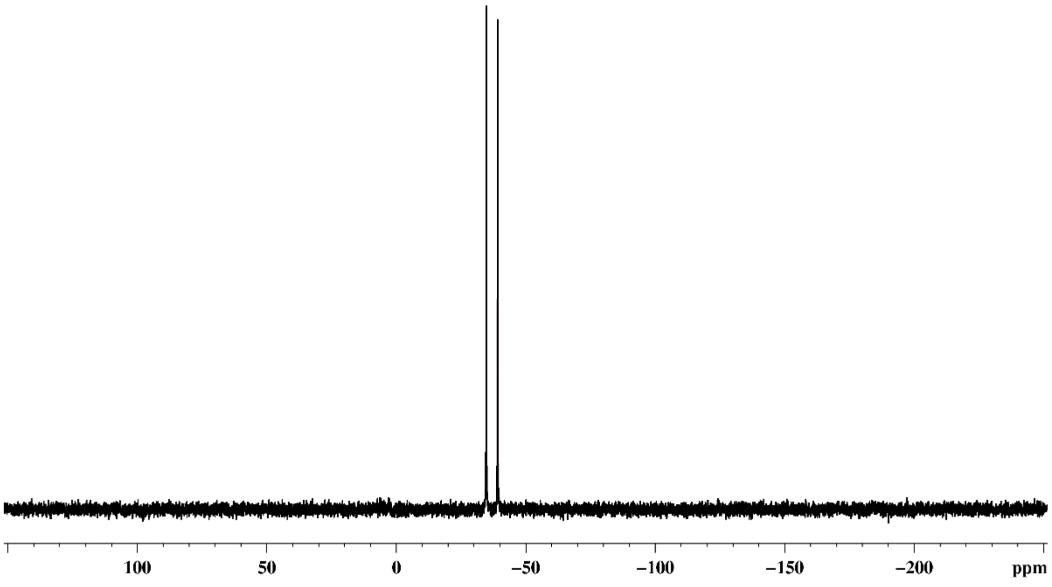

Figure S12. Room temperature ^{31}P NMR spectrum of **3** (C_6D_6).

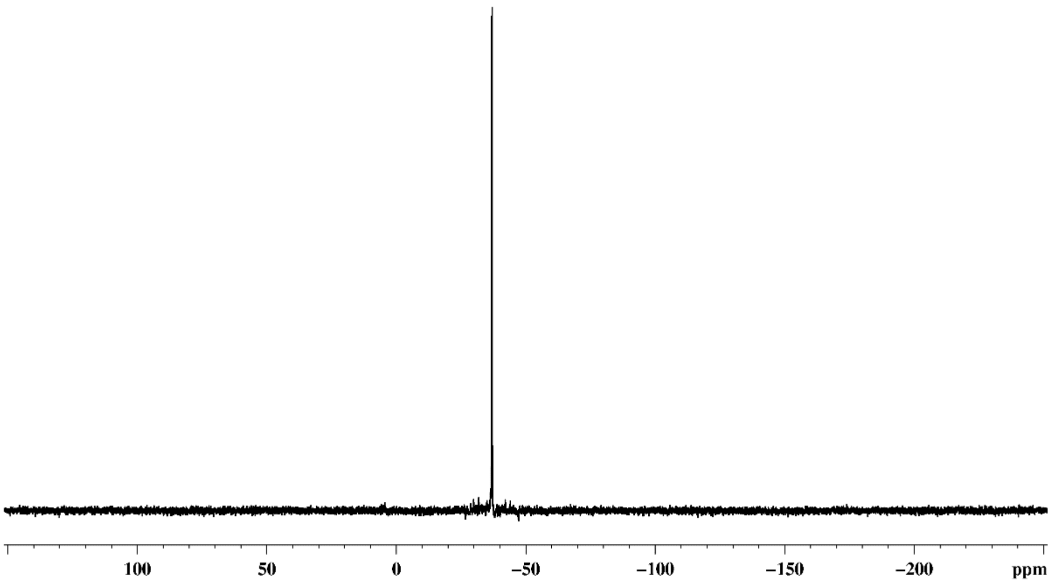

Figure S13. Room temperature $^{31}P\{^1H\}$ NMR spectrum of **3** (C_6D_6).

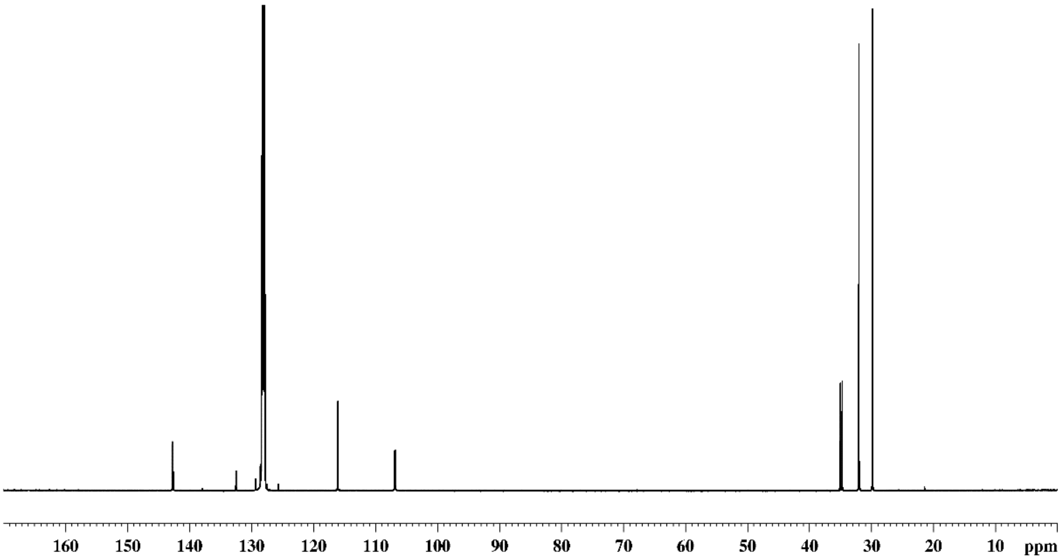

Figure S14. Room temperature $^{13}C\{^1H\}$ NMR spectrum of **3** (C_6D_6).

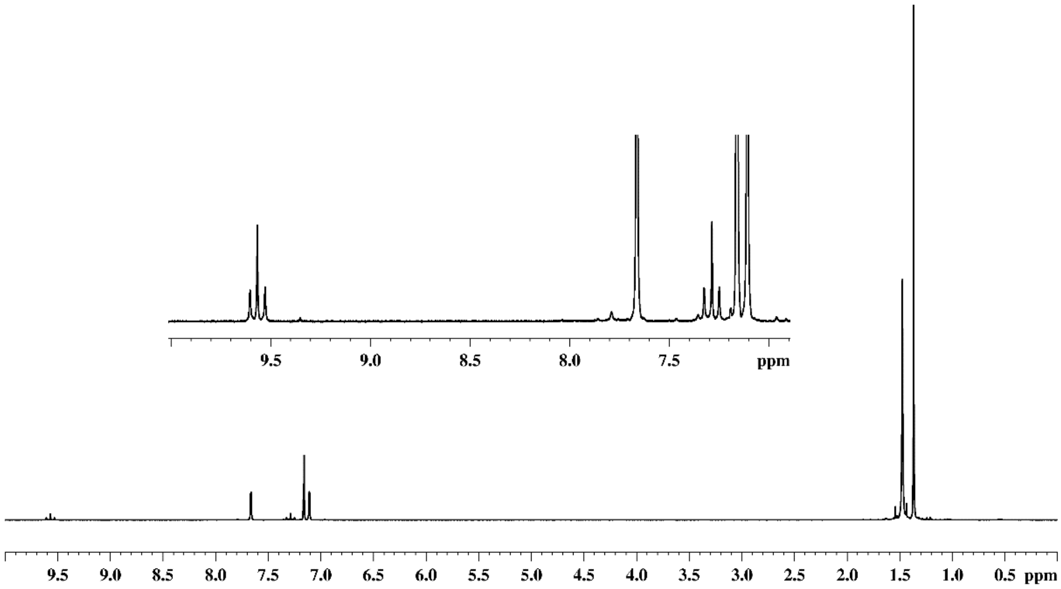

Figure S15. Room temperature ^{1}H NMR spectrum of **4** (C_6D_6).

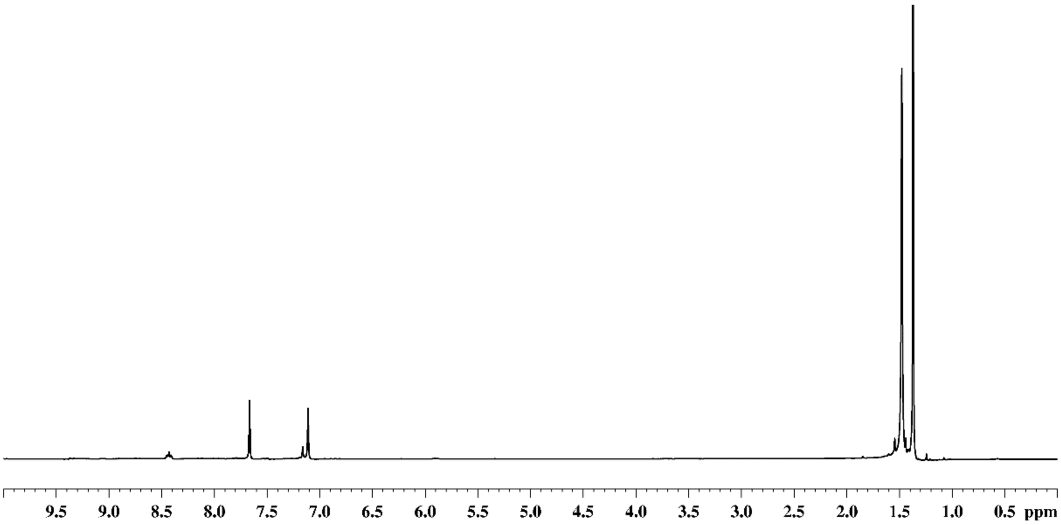

Figure S16. Room temperature $^{1}H\{^{31}P\}$ NMR spectrum of **4** (C_6D_6).

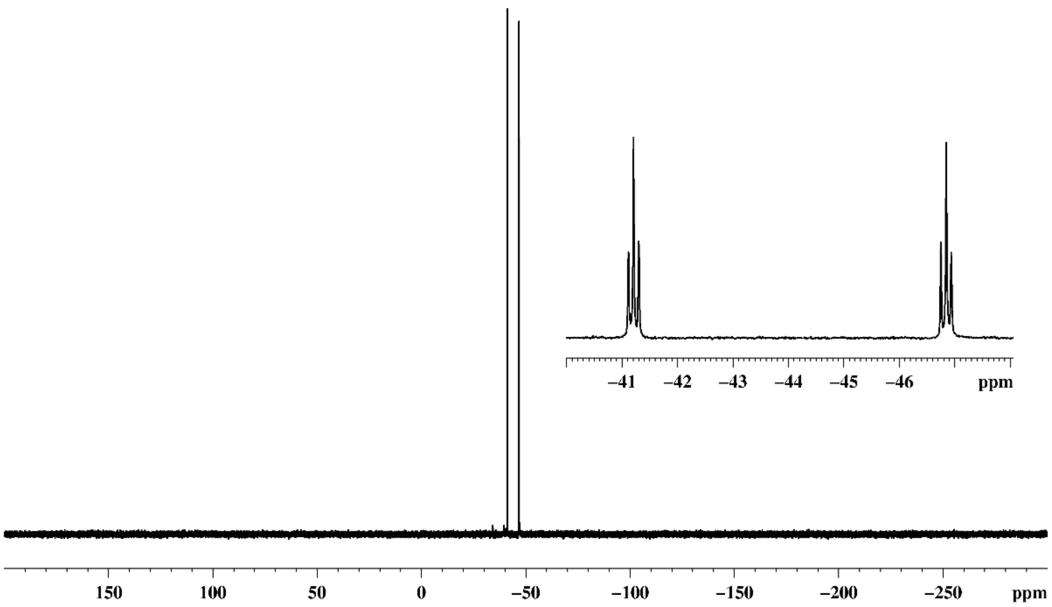

Figure S17. Room temperature ^{31}P NMR spectrum of **4** (C_6D_6).

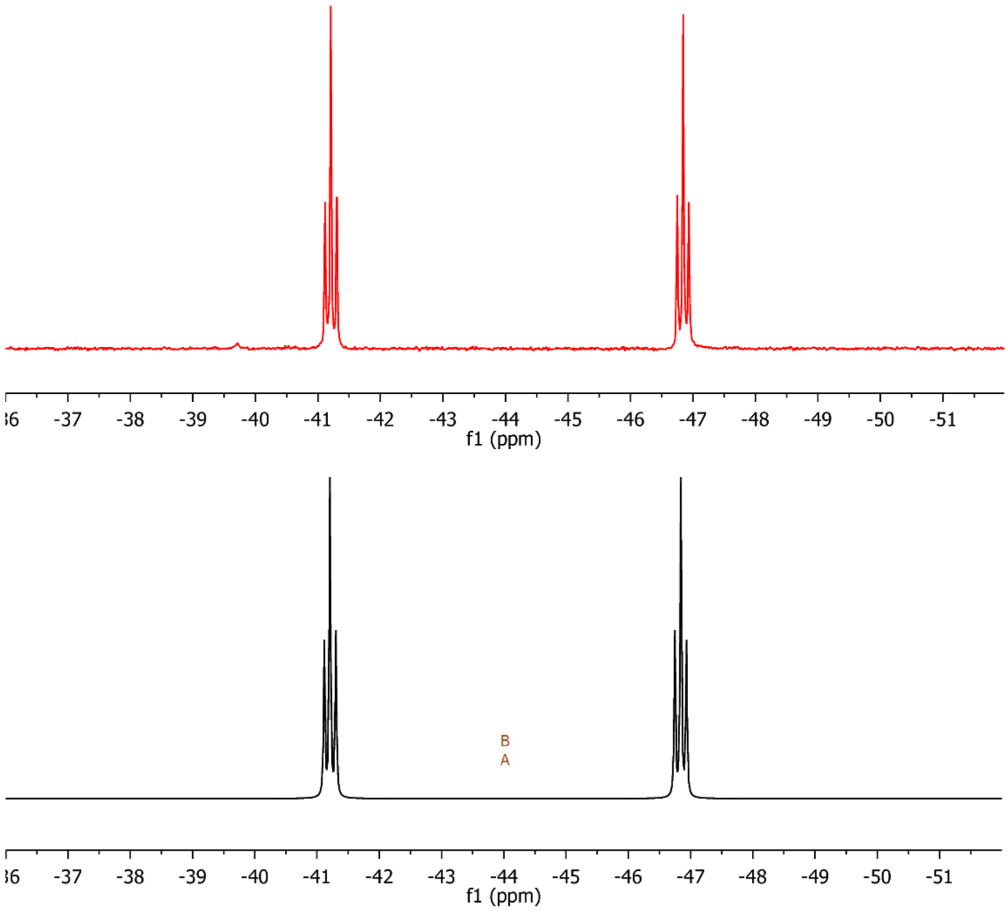

Figure S18. Simulated (bottom/black) and experimental (top/red) ^{31}P NMR spectra of **3**. Simulation performed using the gNMR software.

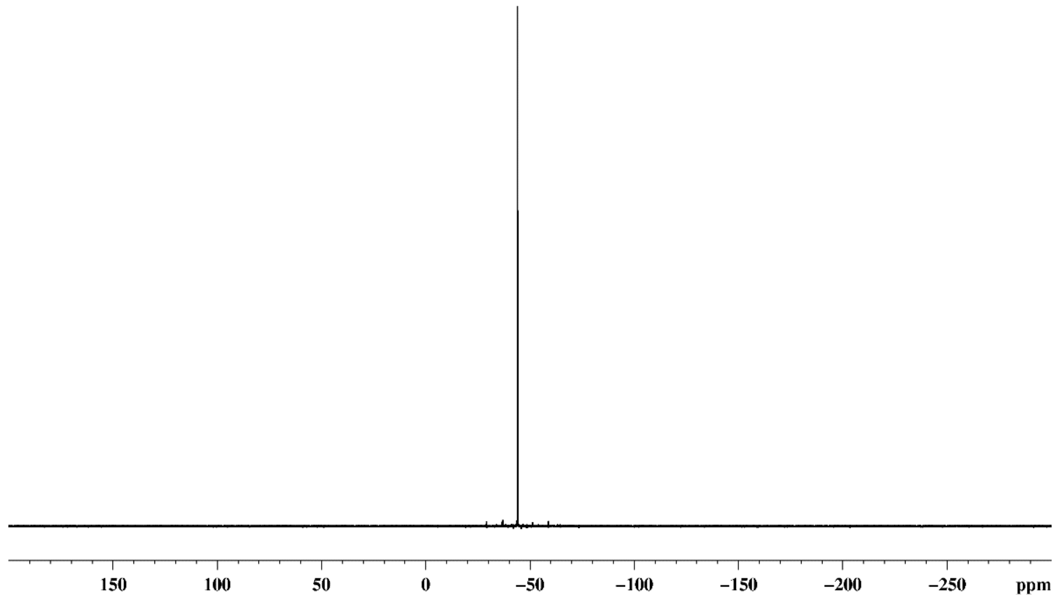

Figure S19. Room temperature $^{31}P\{^{1}H\}$ NMR spectrum of **4** (C_6D_6).

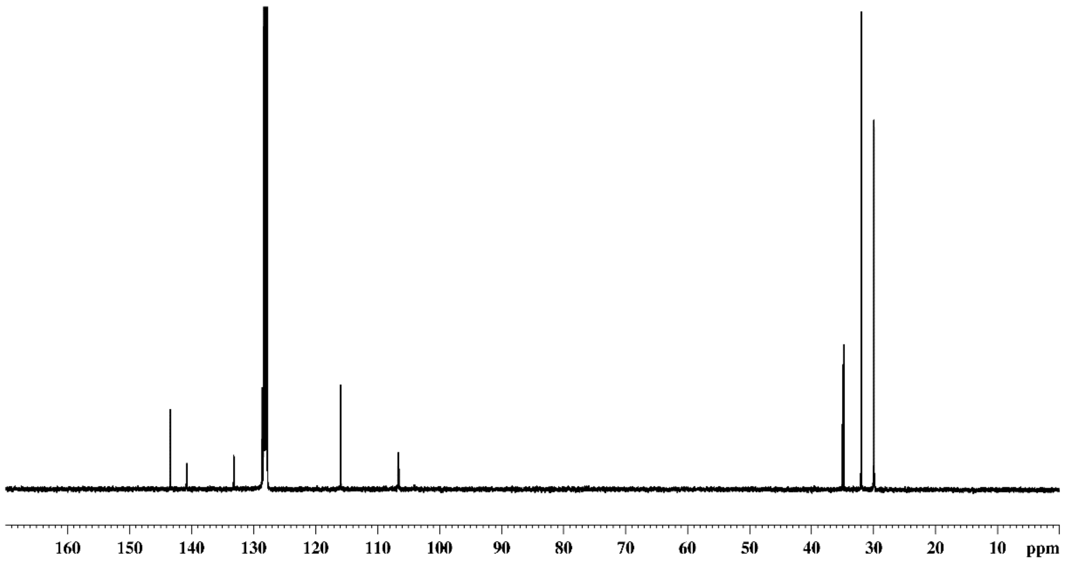

Figure S20. Room temperature $^{13}C\{^{1}H\}$ NMR spectrum of **4** (C_6D_6).

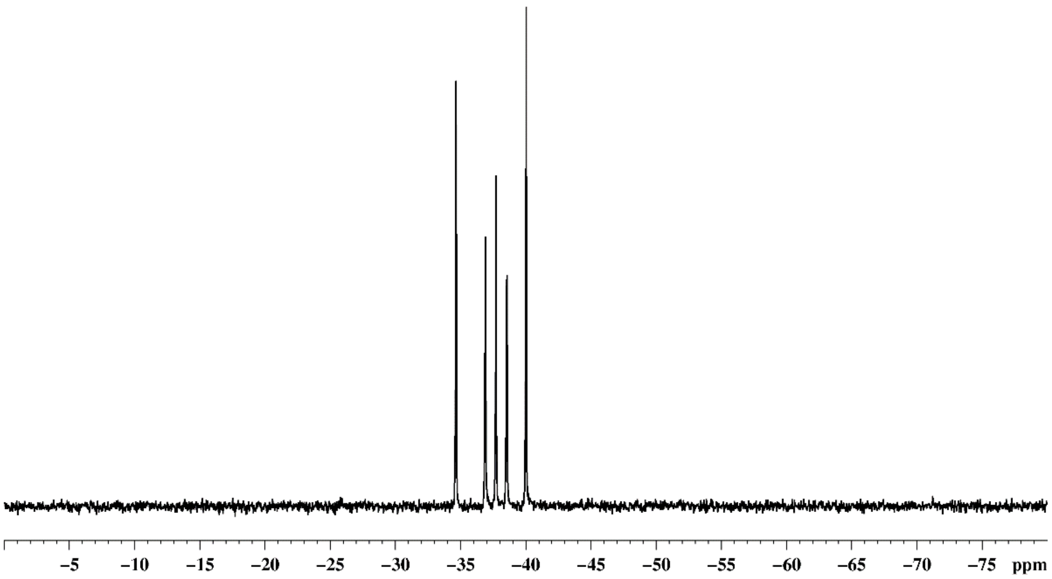

Figure S21. Room temperature ^{31}P NMR spectrum of sample containing a 1:1 stoichiometric ratio of **3** and D_2O after heating to 70 °C (proteo-THF).

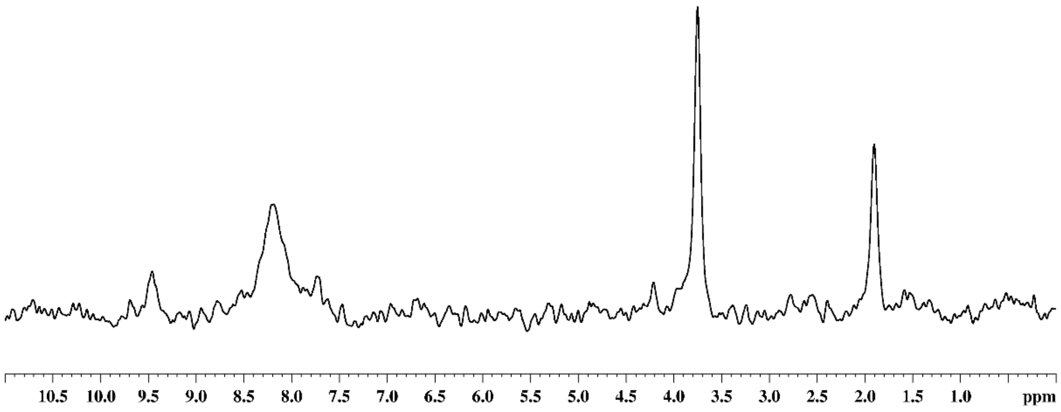

Figure S22. Room temperature ^{2}D NMR spectrum of the reaction between **3** and 1 equivalent of D_2O at room temperature (proteo-THF).

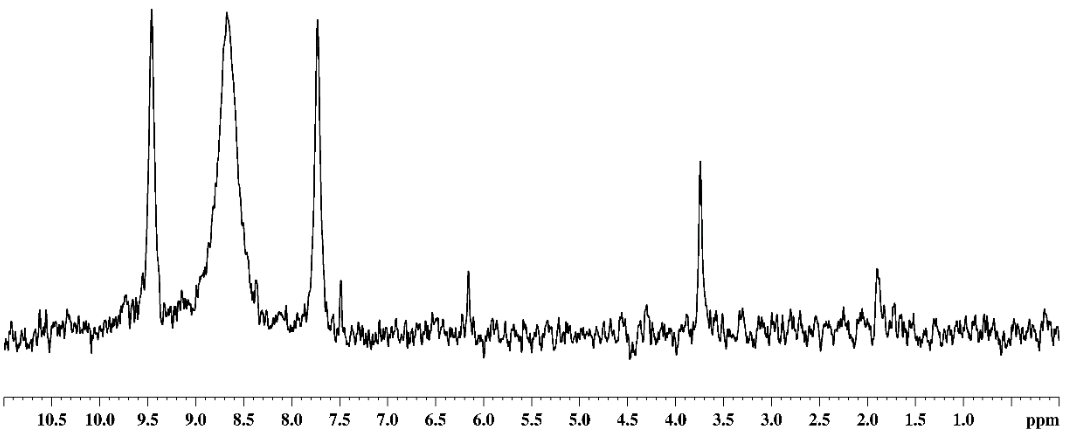

Figure S23. Room temperature ^{2}D NMR spectrum of the reaction between **3** and 1 equivalent of D_2O heated to 70 ^{o}C (proteo-THF).

5. Mass Spectra

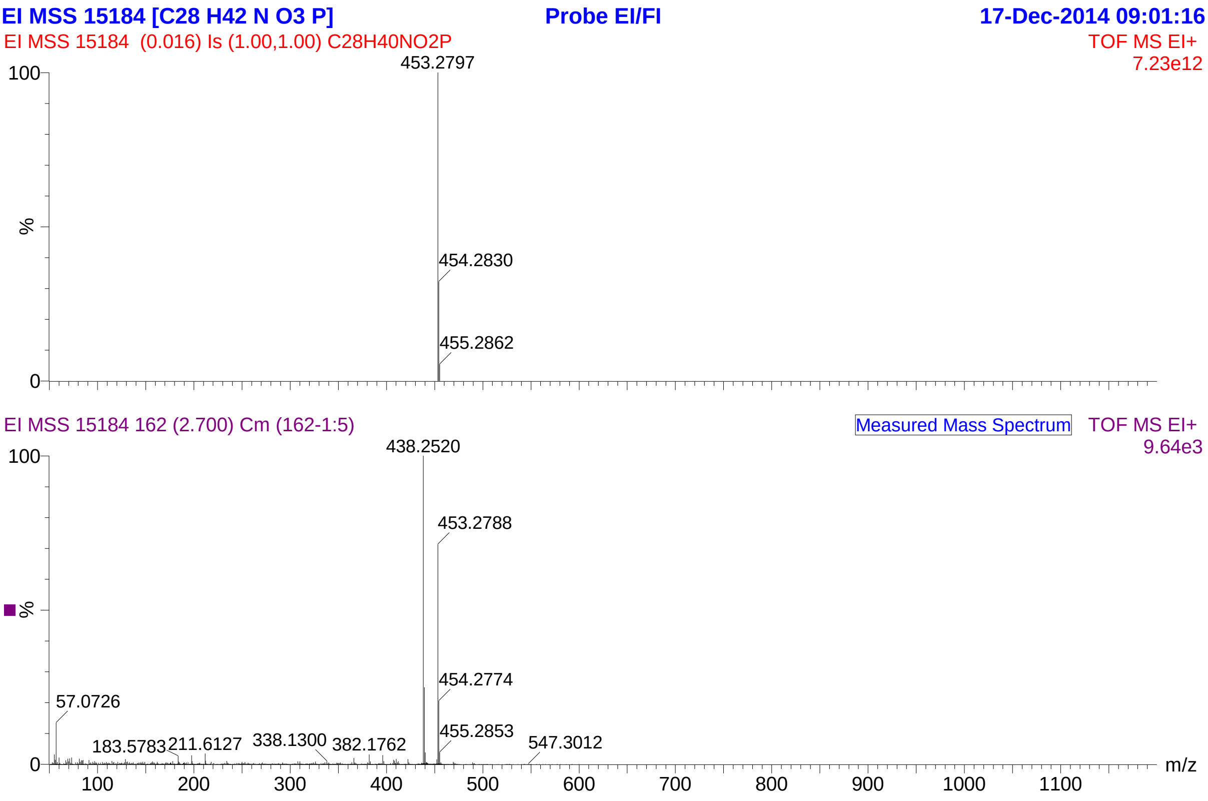

Figure S24. EI-Mass spectrum of **1**.

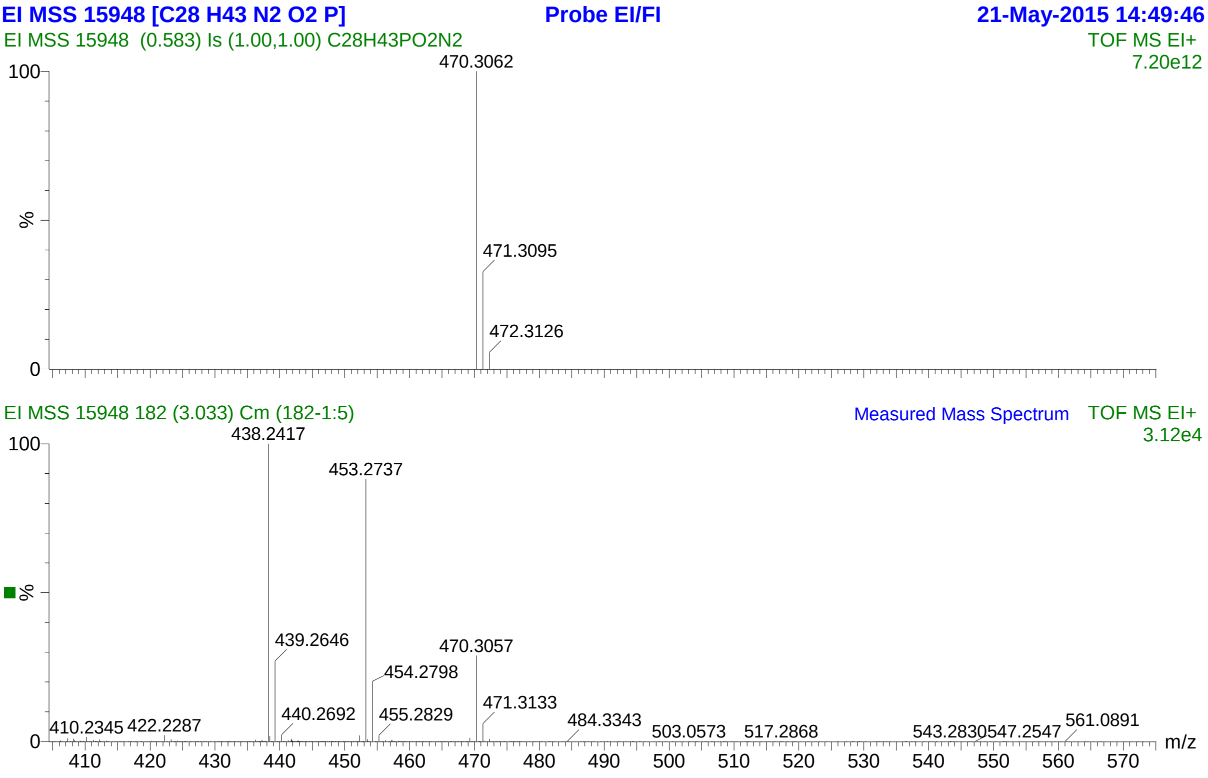

Figure S25. EI-Mass spectrum of **2**.

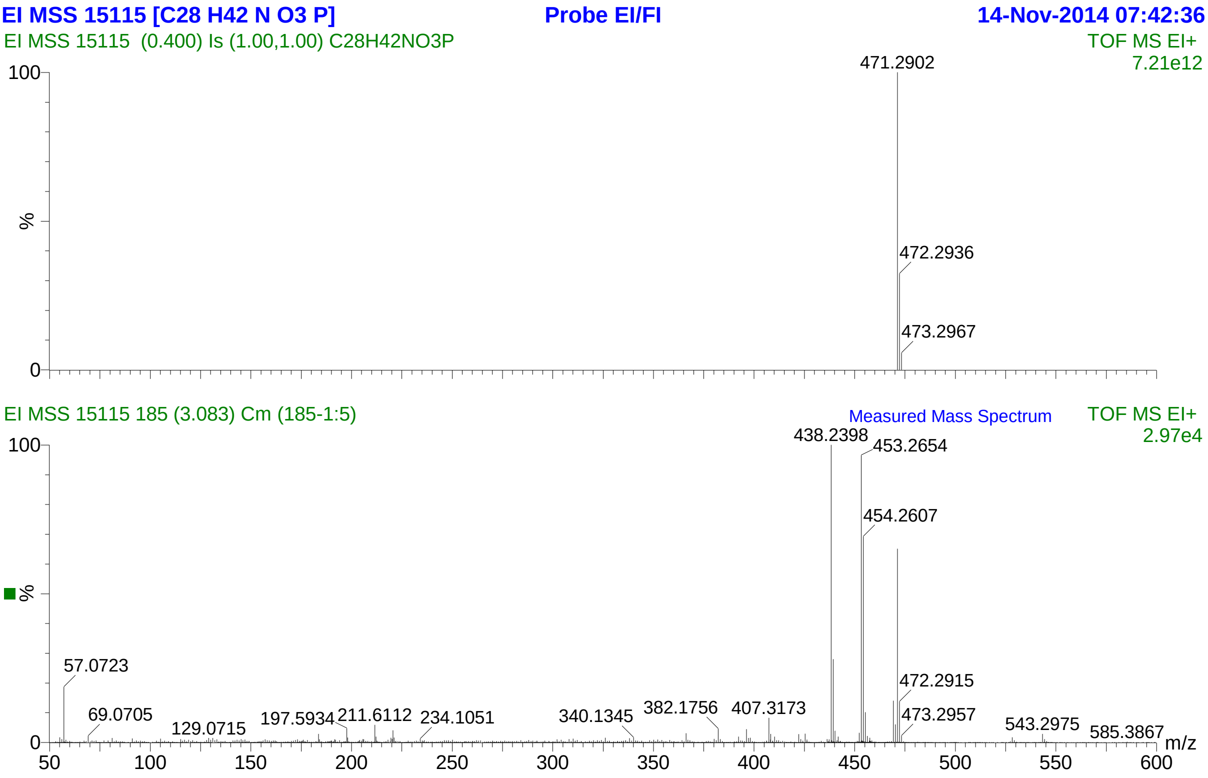

Figure S26. EI-Mass spectrum of **3**.

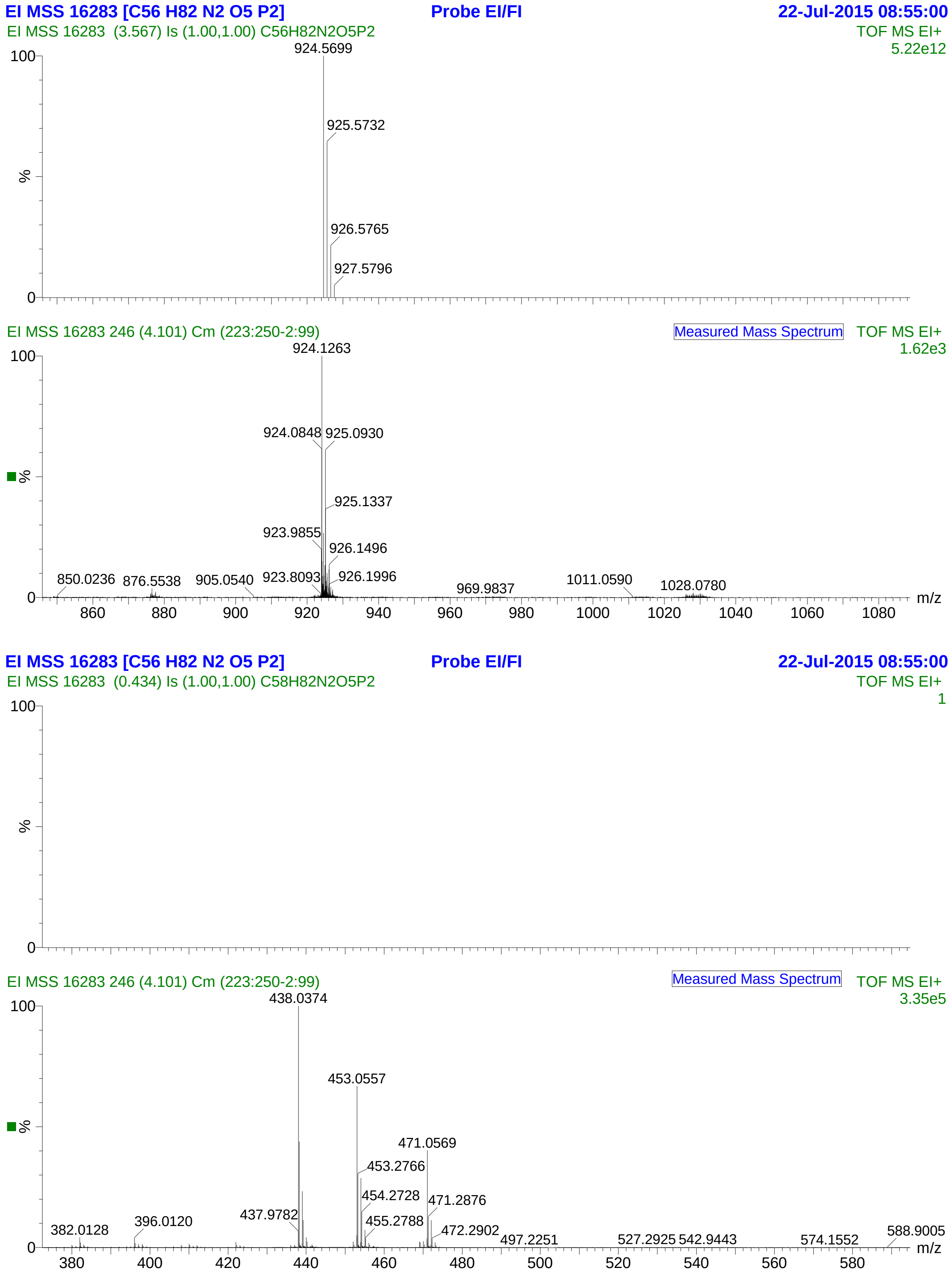

Figure S27. EI-Mass spectrum of **4**.

6. IR Spectra

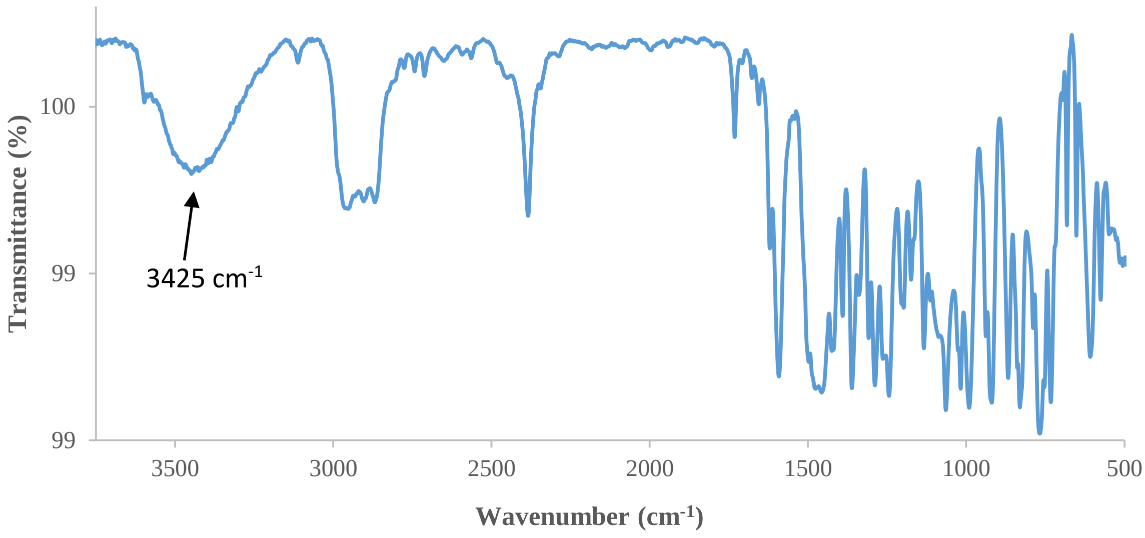

Figure S28. FT-IR spectrum of **3** with O–H vibration labelled (KBr disk).

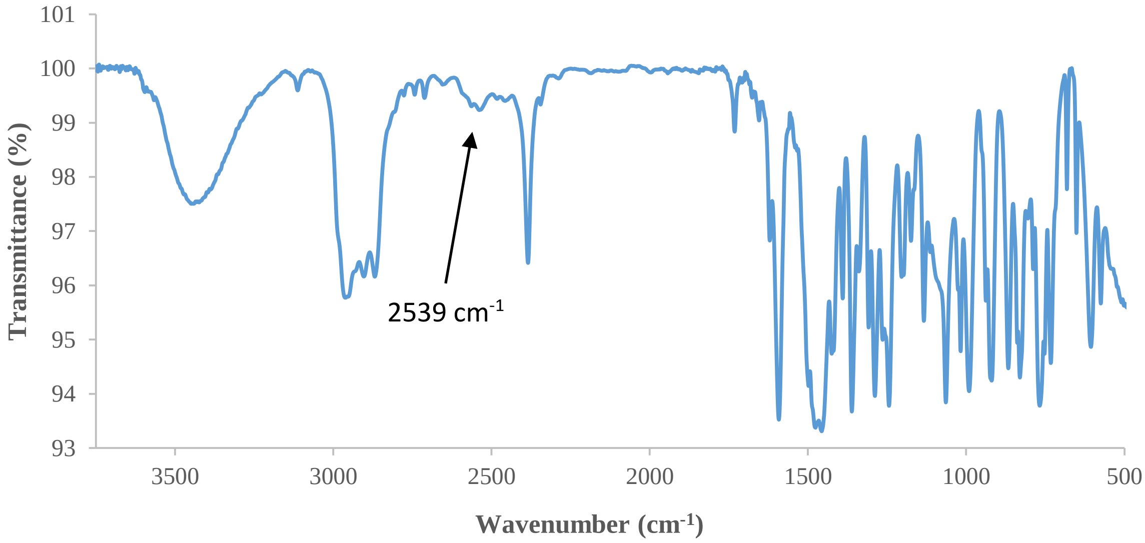

Figure S29. FT-IR spectrum of **3** with 1 eq. of D_2O. O–D Vibration labelled. Sample dried *in vacuo* prior to analysis (KBr disk).

7. References:

[1] P. Chaudhuri, M. Hess, T. Weyhermüller, K. Wieghardt, *Angew. Chem. Int. Ed.* **1999**, *38*, 1095–1098; b) R. A. Zarkesh, J. W. Ziller, A. F. Heyduk, *Angew. Chem. Int. Ed.* **2008**, *47*, 4715–4718.

[2] CrysAlisPro, Agilent Technologies, Version 1.171.35.8.

[3] a) G. M. Sheldrick in SHELXL97, Programs for Crystal Structure Analysis (Release 97-2), Institut für Anorganische Chemie der Universität, Tammanstrasse 4, D-3400 Göttingen, Germany, 1998; b) G. M. Sheldrick, *Acta Crystallogr. Sect. A* **1990**, *46*, 467−473; c) G. M. Sheldrick, *Acta Crystallogr. Sect. A* **2008**, *64*, 112−122.

[4] a) G. te Velde, F. M. Bickelhaupt, E. J. Baerends, C. Fonseca Guerra, S. J. A. van Gisbergen, J. G. Snijders, T. Ziegler, *J. Comput. Chem.* **2001**, *22*, 931−967; b) C. Fonseca Guerra, J. G. Snijders, G. te Velde, E. J. Baerends, *Theor. Chem. Acc.* **1998**, *99*, 391−403; c) ADF2013.01, SCM, Theoretical Chemistry, Vrije Universiteit: Amsterdam, The Netherlands, http://www.scm.com.

[5] A. D. Becke, *J. Chem. Phys.* **1993**, *98*, 5648−5652.

[6] C. Lee, W. Yang, R. G. Parr, *Phys. Rev. B* **1988**, *37*, 785−789.

[7] S. H. Vosko, L. Wilk, M. Nusair, *Can. J. Phys.* **1980**, *58*, 1200−1211.

[8] L. Versluis, T. Ziegler, *J. Chem. Phys.* **1988**, *88*, 322−328.
